# Highly Active and Stable Immobilized Iridium Complexes via Thermochemically Assisted Dangling Oxygen Participation for Electrochemical Oxygen Evolution Reaction

**DOI:** 10.1002/smsc.202500027

**Published:** 2025-05-09

**Authors:** Sang Youn Chae, Myeong Jin Choi, Si Young Lee, Ja Yoon Choi, Dae Won Kim, Je Seung Lee, Eun Duck Park, Jong Suk Yoo, Oh-Shim Joo

**Affiliations:** ^1^ Department of Energy Systems Research Ajou University Suwon 16499 Republic of Korea; ^2^ Ajou Energy Science Research Center Ajou University Suwon 16499 Republic of Korea; ^3^ Department of Chemical Engineering University of Seoul Seoul 02504 Republic of Korea; ^4^ Department of Mechanical Engineering University of Michigan Ann Arbor MI 48109 USA; ^5^ Clean Energy Research Center Korea Institute of Science and Technology Seoul 02792 Republic of Korea; ^6^ Department of Chemistry Kyung Hee University Seoul 02453 Republic of Korea; ^7^ Department of Chemical Engineering Ajou University Suwon 16499 Republic of Korea

**Keywords:** *μ*‐oxo bridges, in situ Raman spectroscopy, lattice oxygen participation mechanism, molecular complexes, oxygen evolution reaction

## Abstract

This study investigates the immobilization of dinuclear iridium‐imidazole complexes onto indium tin oxides for the electrochemical oxygen evolution reaction (OER) in acidic media. The immobilized iridium complexes show exceptional catalytic activity and stability, which are attributed to the facile cleavage of the elongated *μ*—O bonds between the two iridium metal centers. This cleavage leads to the formation of dangling oxygen, which plays a crucial role in facilitating thermochemical water dissociation. O_2_ is released through a dangling oxygen–participated mechanism, accompanied by the regeneration of the *μ*—O bonds. This unique OER mechanism, possibly specific to immobilized (strained) molecular catalysts, resembles the lattice oxygen participation mechanism reported for unstable oxides, but with the advantage of high stability in acidic media. This study not only identifies a new mechanism but can also inform the design of immobilized molecular catalysts with enhanced performance.

## Graphical abstract

Dinuclear iridium complexes immobilized on ITO surfaces enable highly efficient and stable oxygen evolution in acidic media. Through a novel mechanism involving the reversible cleavage/regeneration of distorted μ‐oxo bridges, the catalysts facilitate the thermochemically assisted dangling oxygen participation mechanism. This study unveils a unique and durable pathway for oxygen evolution, offering insights for next‐generation molecular electrocatalyst design.

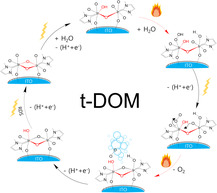

## 1 Introduction

Electrochemical water oxidation plays a critical role in energy conversion as an electron source for various processes.^[^ ^1^ ^]^ proton exchange membrane (PEM) electrolyzers have been widely used in water electrolysis; however, many oxygen evolution catalysts show limited stability, especially in acidic media within PEM electrolyzers.^[^ ^2^ ^]^ Metal oxides, including rutiles and perovskites, are commonly employed electrocatalysts for the oxygen evolution reaction (OER), with iridium‐based oxide materials being among the most active catalysts.^[^ ^3^ ^]^

**Figure 1:**
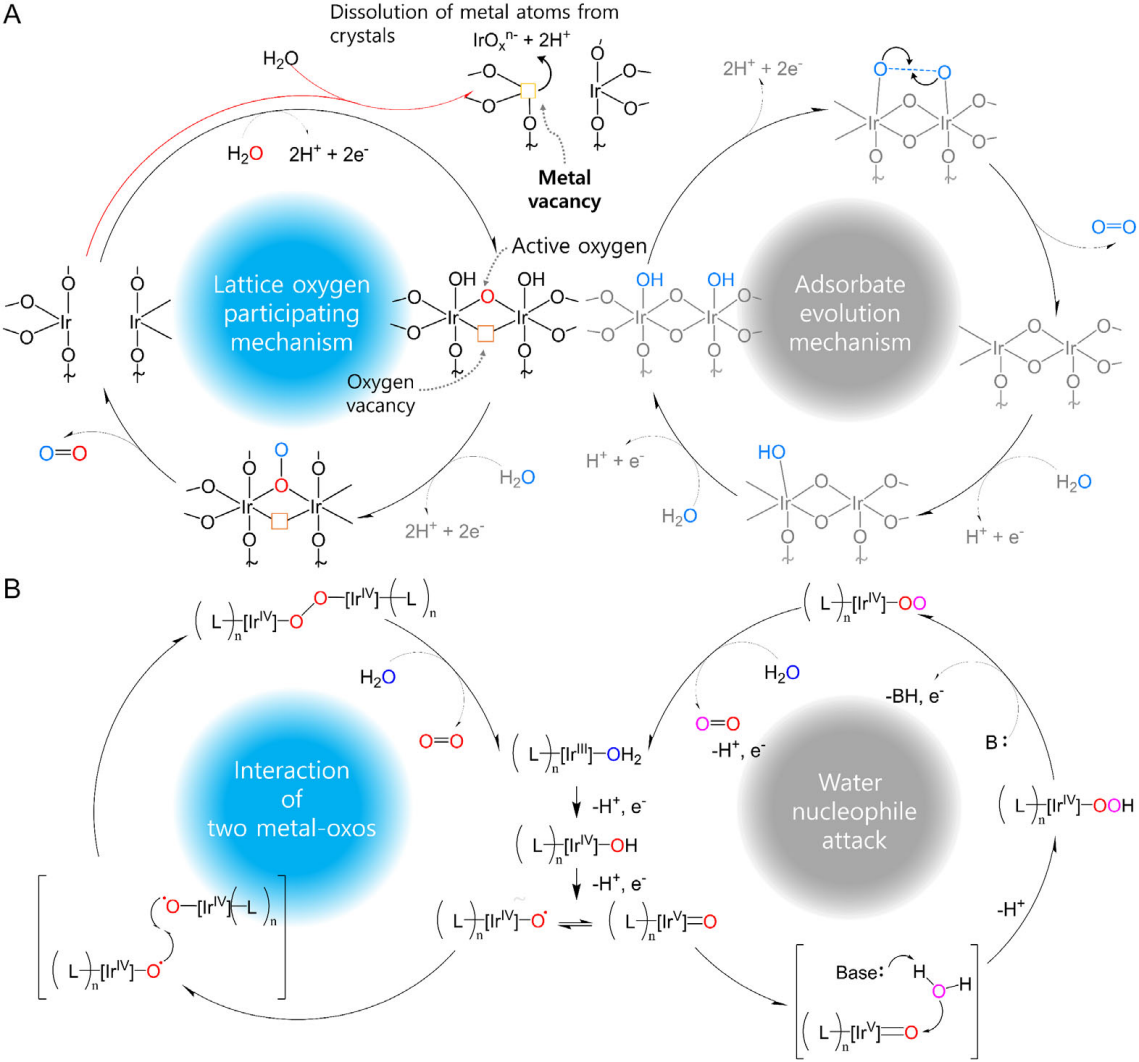
Known OER mechanism of metal oxides and molecular complexes. A) Iridium oxides in a heterogeneous system. B) Ir complexes in a homogeneous system.

Although the OER mechanism for metal oxides is under debate, two distinct hypotheses have been proposed: 1) the adsorbate evolution mechanism (AEM) involving stepwise electro‐oxidation of adsorbed water to oxygen gas on metal sites; and 2) the lattice oxygen participation mechanism (LOM) where lattice oxygens directly participate in the formation of oxygen gas (**Figure** **1**a).^[^ ^4^, ^5^ ^]^ Previous theoretical studies suggested that LOM leads to superior OER activity compared to AEM. However, LOM is more susceptible to catalytic instability and is preferred to AEM on relatively unstable (highly reducible) oxides.^[^ ^6^, ^7^, ^8^ ^]^ For example, IrO_2_–*δ* and Ba_0.5_Sr_0.5_Co_0.8_Fe_0.2_O_3–*δ*_ (BSCF), which exhibit high OER activity through LOM, encounter deactivation issues including the dissolution of metal atoms into acidic electrolytes.^[^ ^9^, ^10^, ^11^ ^]^ Thus, a trade‐off between catalytic activity and stability is inevitable for the OER via LOM on metal oxides.^[^ ^6^ ^]^ The development of both highly active and stable electrocatalysts, especially in acidic media, remains an important research goal.

Metal–ligand molecular complexes are promising candidates as catalysts with high activity and stability for the OER. These complex catalysts have a high turnover frequency (TOF) for water‐splitting reactions, and they also have the advantage of activity tuning through ligand modification.^[^ ^12^, ^13^, ^14^, ^15^, ^16^ ^]^ Similar to metal oxide electrocatalysts, the OER mechanism in the metal complex can also be distinguished by two different mechanisms (Figure 1b). One is the water nucleophile attack (WNA), in which water molecules attack a single active site and are further oxidized into O_2_. The other one is the interaction of two metal–oxos (I2M), in which two intermediates in two different active sites form an M—O—O—M bond.^[^ ^17^, ^18^ ^]^ WNA metal complex catalysts tend to be slower than I2M metal complex catalysts because the formation of electrophile metal–oxo requires higher potential than the M–*μ*‐O–M bridges in an I2M catalyst.^[^ ^18^, ^19^ ^]^ However, although the TOF of the metal complex is sufficiently high in a homogeneous water catalysis system, the reported activity of metal complex electrocatalysts in heterogeneous systems is significantly inferior to that of metal oxide catalysts. At similar overpotential, immobilized metal complex electrocatalysts show 10–100 times less reaction rates (i.e., current density).^[^ ^2^, ^3^, ^20^, ^21^, ^22^, ^23^, ^24^, ^25^, ^26^, ^27^, ^28^, ^29^, ^30^, ^31^, ^32^, ^33^ ^]^ The main reason for the inferior activity is that most of the reported studies for the immobilized metal complex are on mononuclear complexes, and the I2M mechanism is restricted to surface‐immobilized conditions.^[^ ^34^ ^]^

This reduced activity can be attributed, in part, to the prevalent focus on immobilizing mononuclear complexes, whereas the preferred I2M mechanism is better suited for dinuclear motifs.^[^ ^34^ ^]^ The utilization of dinuclear molecular structures for immobilization provides a promising solution, eliminating the need for in situ metal–oxo–metal bridge formation during OER.^[^ ^34^, ^35^ ^]^ A dinuclear structure can be helpful to circumvent this restriction because it do not require a bimolecular formation step. More importantly, the molecule does not have defect sites unlike imperfect solid crystals. Therefore, the defect‐free structure of molecular complexes is of a less concern in terms of the dissolution of metal atoms during the reaction because the ligands are strongly bonded with the central metal atoms.

In this study, we synthesized and immobilized dinuclear Ir‐imidazole complexes ([Ir(dmimd)(OH)(H_2_O)_2_(*μ*‐O)]_2_^2+^, dmimd =1,3‐dimethyl imidazole) onto an indium tin oxide (ITO) nanoporous film for OER in acidic media. Interestingly, the immobilized dinuclear Ir‐imidazole complexes facilitated OER through a mechanism similar to the LOM for metal oxides. Both in operando Raman spectroscopy and density functional theory (DFT) calculations support the notion that this new mechanism involves the direct participation of dangling oxygen resulting from the cleavage of *μ*‐O bridges in the generation of oxygen gas.

## 2 Results and Discussion

### 2.1 Electrocatalytic Activities of Dinuclear Molecular Complexes

**Figure 2:**
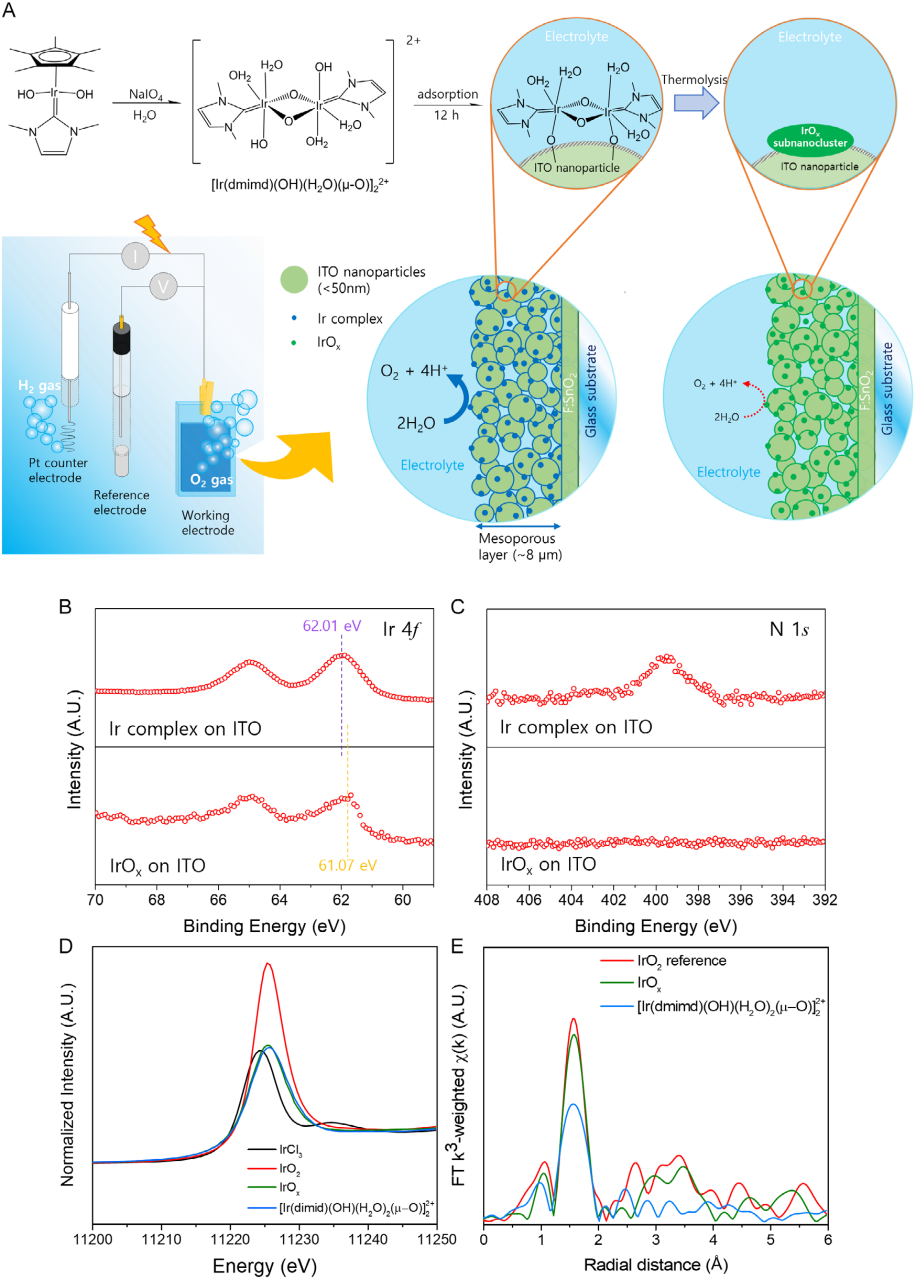
A scheme for the molecular structure of immobilized Ir complexes for OER and its XPS/XAS characterization. A) A scheme for the synthesis and molecular structure of [Ir(dmimd)(OH)(H_2_O)_2_(*μ*‐O)]_2_^2+^, and the illustrations of a setup for electrochemical water oxidation and the nanostructure for a mesoporous working electrode. IrO_*x*_–ITO was also prepared from Ir‐complex_(ad)_/ITO films to compare the catalytic activity with the same amount of Ir. B,C) Ir 4*f* and N 1*s* XPS spectra of Ir complex and IrO_*x*_ adsorbed on ITO nanoparticles. D,E) XANES and EXAFS spectra of Ir complexes and IrO_*x*_ adsorbed on ITO nanoparticles.

The synthesized Ir‐imidazole dinuclear complexes were immobilized on a nanoporous ITO layer deposited on a fluorine‐doped tin oxide (FTO) glass substrate, resulting in an Ir complex_(ad)_/ITO/FTO structure (**Figure** **2**). Extensive characterization was conducted, revealing distinct features with ^1^H nuclear magnetic resonance spectroscopy, Raman spectroscopy, Fourier‐transformed infrared (FTIR) spectroscopy, and transmittance electron microscopy (TEM).

Two peaks of ^1^H nuclear magnetic resonance (NMR) spectra corresponding to CH and CH_3_ of dimethyl imidazole ligands were observed at ≈7 and 4 ppm from Ir^III^Cp*(dmimd)Cl_2_ and Ir^III^Cp*(dmimd)(OH)_2_ (complexes 1 and 2, Figure S1, Supporting Information), respectively. CH_3_ peaks of Cp* in each complex were observed at ≈1.7 ppm. It is known that the peaks of dimethyl imidazole can be observed at ≈3*δ* for a single bond C—H, ≈2.5*δ* for N—H, and ≈4*δ* for a double bond C=H. This shift in *δ* is due to electron deficiency by the binding of the Ir^III^ T_d_ configuration. For the catalyst—[Ir(dmimd)(OH)(H_2_O)_2_(*μ*‐O)]_2_^2+^ (complex 3, Figure S2, Supporting Information) prepared by dimerization with the di‐oxo bridge formation of complex 2, ^1^H NMR was performed. In this reaction, the Cp* ligand is dissociated and oxidized by an oxidant.

We believe that only the Cp* ligand leaves from the Ir metal, and H_2_O and *μ*–O ligands are formed during this reaction compared to the dimerization reaction of Ir mononuclear complexes.^[^ ^14^, ^22^ ^]^ Ir has an oxidation state of +4 and a *d* ^5^ electronic configuration. Although the oxidation state of Ir is +4, it has been reported that chemicals with bis*(μ*‐oxo) bridge ligands have diamagnetic properties owing to antiferromagnetic coupling.^[^ ^36^, ^37^, ^38^, ^39^, ^40^, ^41^ ^]^ As a result, the NMR peaks corresponding to the dimethyl imidazole (at ≈2*δ* for N—H and ≈3*δ* for the double bond C=H) in complex 3 are slightly shifted by the Ir—L bond.

The peaks of the single C—H bond were not observed because of the overlapping peak with H_2_O (≈4.7*δ*). This result is in accordance with the ^13^C NMR results of complex 2 (≈120*δ* for Cp*–C, ≈85*δ* for C‐ of imidazole, ≈36*δ* for CH_3_‐ of imidazole, ≈10*δ* for Cp*–CH_3_) and complex 3 (≈180*δ* for Cp*–C, ≈160*δ* for C‐ of imidazole, ≈25*δ* for CH_3_‐ of imidazole, ≈20*δ* for Cp*–CH_3_), in which a similar peak shifting was observed (Figure S3, Supporting Information). Such peak shifting has also been reported for different Ir mononuclear and dinuclear complexes.^[^ ^14^ ^]^

In FTIR analysis, any signals related to the Ir complex were not revealed after immobilization of Ir complexes on an 8 μm nanoporous ITO film (thickness of ITO layers was confirmed by scanning electron microscopy, Figure S4, Supporting Information); however, ex situ Raman spectroscopy detected two peaks indicating the presence of *μ*–O bridges (484 and 768 cm^−1^) and the C—N vibrational mode in dimethyl imidazole (1360 cm^−1^) (Figure S5, Supporting Information). High‐resolution transmission electron microscopy (HR‐TEM) could not be used to examine the Ir complex immobilized on the ITO lattice due to decomposition caused by the incident electron beam (Figure S6, Supporting Information). Similar observations of metal complex decomposition under electron beam exposure have been reported previously.^[^ ^21^, ^22^ ^]^

X‐ray photoelectron spectroscopy (XPS) analysis showed different binding energies for Ir 4*f* between Ir complexes/ITO and thermally oxidized Ir complexes (IrO_*x*_)/ITO films. Furthermore, the presence of nitrogen atoms was observed in the Ir complexes but absent in IrO_*x*_ (Figure 2b,c). By utilizing X‐ray absorption near edge structure (XANES) and Fourier‐transformed *k* ^3^‐weighted extended X‐ray absorption fine structure (EXAFS) analysis (Figure 2d,e), it was inferred that the coordination number of Ir centers in the complex was lower compared to fully coordinated IrO_2_. Specifically, the signals indicated a decrease in the coordination number of oxygen around Ir metals in the first shell, with the order being IrO_2_ > IrO_*x*_ > Ir complexes. The oxidation state of Ir for each sample was found to decrease in the following order: IrO_2_ > Ir complexes ≥ IrO_*x*_ > IrCl_3_ (Figure S7, Supporting Information).

**Figure 3:**
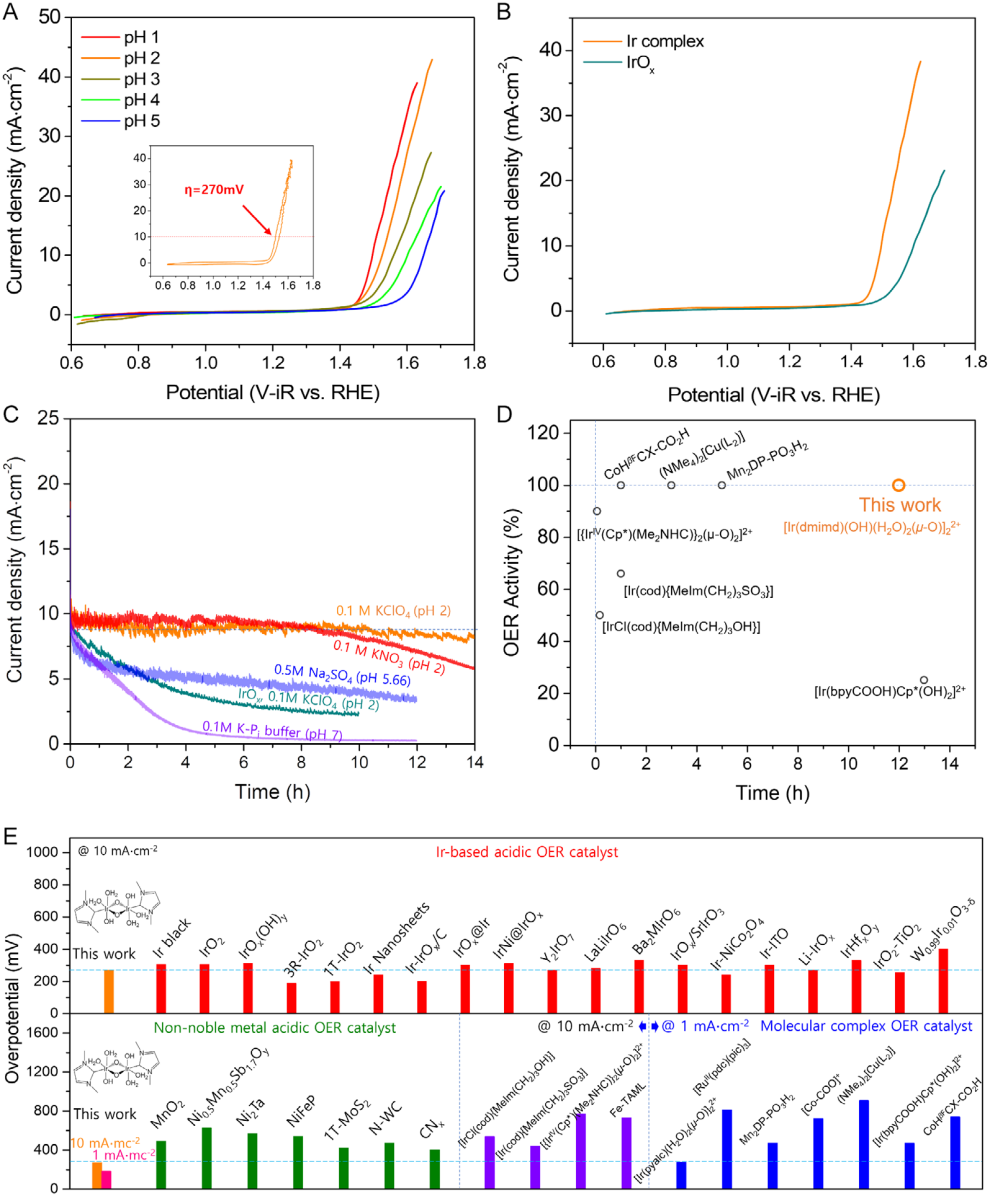
OER activity comparison of Ir complexes and other electrocatalysts. A) Cyclic voltammetry results of an [Ir(dmimd)(OH)(H_2_O)_2_(*μ*‐O)]_2_^2+^ adsorbed ITO film and IrO_*x*_ on an ITO film in a 0.1 M KClO_4_ electrolyte. The inset graph in (a) shows the overpotential in the 0.1 M KClO_4_ (pH 2) condition. B) Cyclic voltammetry comparison of Ir complexes and IrO_*x*_. C) Stability tests for Ir complexes and IrO_*x*_ on ITO nanoporous films on FTO substrates in various electrolytes. D) Stability comparison of immobilized molecular complexes with reported complexes. The time and OER activity indicate testing time for stability and ratio of initial/final current density values after stability tests. E) Overpotential comparison with Ir‐based, non‐noble metal‐based, and molecular complex electrocatalysts for OER.

Electrochemical OER experiments were performed using an Ir complex_(ad)_/ITO electrode under various salts and pH conditions (**Figure** **3**a–c and Figure S8, Supporting Information). Among the tested electrolytes, KClO_4_ (pH 2) exhibited the highest OER activity and superior overall performance. However, there was a substantial reduction in activity with increasing pH, primarily due to the detachment of Ir complexes from the ITO surface. Nonetheless, our electrode delivered outstanding results, achieving low overpotential of only 184 mV at the current density (*j*) of 1 mA cm^−2^ and 270 mV at 10 mA cm^−2^ (Figure 3b). The Faradaic efficiency for O_2_ evolution with the Ir complex_(ad)_/ITO/FTO electrode exceeded 95% (Figure S9, Supporting Information), placing it among the top‐performing immobilized molecular complexes. The Tafel slope value of ≈120 mV dec^−1^ indicated that the rate‐determining step involved a one‐electron transfer (Figure S10, Supporting Information).^[^ ^42^ ^]^ The TOF for the immobilized Ir complexes was 0.067 and 0.133 mol s^−1^ mol^−1^ at 10 and 20 mA cm^−2^, respectively (Figure S11, Supporting Information). These values are highly comparable to those of IrO_*x*_ and other transition‐metal oxides (0.001–0.1).^[^ ^43^ ^]^ In addition, the mass activity of the Ir complex (Ir mass) was estimated to be 69.42 A g^−1^ at 1.5*V*_RHE_ (270 mV overpotential) in 0.1 M KClO_4_ (pH 2) (Figure S12, Supporting Information). The activity of the immobilized Ir complexes was also comparable with many state‐of‐the‐art inorganic materials and molecular catalysts (Figure 3e and Table S1, Supporting Information).^[^ ^2^, ^3^, ^20^, ^21^, ^22^, ^23^, ^24^, ^25^, ^26^, ^27^, ^28^, ^29^, ^30^, ^31^, ^32^, ^33^, ^35^ ^]^

We further evaluated the electrocatalytic performance by comparing the immobilized Ir complexes with IrO_*x*_/ITO, which was obtained by annealing Ir complex_(ad)_/ITO to maintain the same Ir content as the immobilized Ir complexes. Higher annealing temperatures had more detrimental effects on OER activity (Figure S13a, Supporting Information) and led to a decrease in the surface area of ITO nanoparticles due to thermal aggregation. Therefore, the surface area of Ir complexes‐ or IrO_*x*_‐loaded electrodes was estimated using electrochemical surface area (ECSA) measurements (Figure S13b–f, Supporting Information). Indeed, annealing resulted in the decreased ECSA of the electrodes. However, even when accounting for ECSA, IrO_*x*_ still exhibited inferior OER activity compared to the Ir complex (Figure S13g, Supporting Information). While no IrO_*x*_ nanoparticles were observed using TEM, energy‐dispersive spectroscopy (EDS) analysis confirmed the presence of iridium species after annealing (Figure S14, Supporting Information). These findings suggest that the Ir complexes decomposed and persisted as Ir‐O dimers or IrO_*x*_ sub‐nanoclusters, consistent with previous reports.^[^ ^44^ ^]^ IrO_*x*_/ITO exhibited significantly higher overpotential (375 mV at 10 mA cm^−2^) compared to the immobilized Ir complexes.

Ensuring catalytic stability is equally crucial to achieving high catalytic activity. Stability tests were conducted under different salts and pH conditions to investigate the effects of electrolytes (**F**igure 3c,d). The bonding between ITO and Ir complexes occurs through the protonation of hydroxyl groups and it is reversible in high pH solvents (in this case, the electrolyte).^[^ ^22^, ^45^, ^46^ ^]^ Consequently, electrode activity rapidly decreased at pH > 5 (sodium sulfate, pH 5.43; phosphate buffer electrolyte, pH 7). The immobilized Ir complexes exhibited remarkable stability during OER in a KClO_4_ electrolyte (pH 2) for 12 h, retaining over 90% of the initial current. This stability outperforms many other immobilized molecular and conventional heterogeneous catalysts, as shown in Table S1, Supporting Information. The enhanced stability is likely due to the stabilization of the adsorbed Ir complexes by ClO_4_^−^ ions. It is worth noting that the stability of the Ir complexes is influenced by anions, even when the electrolyte pH remains unchanged. The Ir complex‐adsorbed electrode displayed superior stability in KClO_4_ compared to KNO_3_, implying that ClO_4_^−^ ions contribute to stabilizing the Ir complex adsorption. In contrast, phosphate anions may promote the detachment of Ir complexes from the ITO nanoparticle surface, as indicated by the rapid decrease in current density observed during the first cyclic voltammetry scan. Beyond the detachment of Ir complexes or anion effects, another factor in deactivation is the dissolution of the ITO support in the acidic electrolyte,^[^ ^47^ ^]^ as XPS analysis revealed significant In dissolution from ITO after the stability test (Table S2, Supporting Information). These results demonstrate exceptional stability, especially considering the high operating current density and low overpotential (Figure 3d,e).

**Figure 4:**
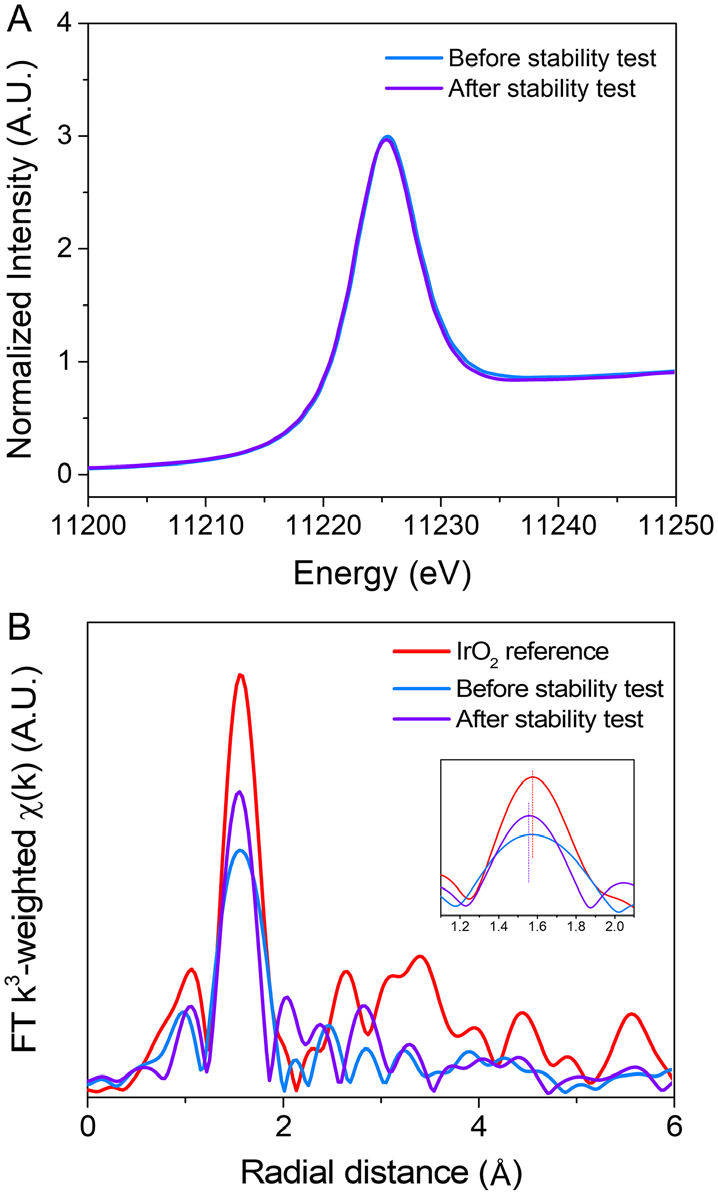
XAS results of Ir complexes. A) XANES spectra of Ir complexes before and after stability tests. B) EXAFS spectra of Ir complexes before and after stability tests.

XPS was conducted to examine the chemical states before and after the test (Figure S15, Supporting Information). The Ir 4*f* binding energy after the electrochemical test is only slightly changed compared to that of the as‐adsorbed state. Additionally, there was no shift in the N 1*s* and C 1*s* binding energy peaks of the imidazole ligands (Figure S15 and S16, Supporting Information), indicating that Ir^4+^ remained bonded with the dmimd ligands. X‐ray absorption spectroscopy (XAS) of the Ir complexes before and after the test revealed only minor changes in the EXAFS signals (**Figure** **4**). The oxidation state of Ir metal centers was comparable before and after the test, based on the XANES spectra (3.45 and 3.22, respectively). The slight difference in oxidation may be due to the incomplete recovery of the oxo‐bridge in unsolvated conditions. However, this difference does not indicate the complete decomposition of the Ir complex into IrO_*x*_ or IrO_2_. This suggests that the Ir complexes maintained their coordination number during the OER process.

In contrast, IrO_*x*_ exhibited a significant decrease to ≈25% of the initial current density after 8 h. The initial Ir 4*f* binding energy (61.07 eV) increased to 62.42 eV in IrO_*x*_ following the test (Figure S17, Supporting Information), indicating that the state of Ir changed from O‐deficient IrO_*x*_ via LOM.^[^ ^48^ ^]^ The lattice oxygen in IrO_*x*_ may contribute to irreversible oxygen‐iridium bond breakage during LOM.^[^ ^6^, ^9^, ^48^ ^]^

The stability of metal–organic complexes often decreases under anodic conditions, leading to their decomposition into metal oxides.^[^ ^49^, ^50^, ^51^, ^52^ ^]^ After the stability test of Ir complex_(ad)_/ITO electrodes, ≈1 nm nanoparticles were observed on the ITO particles (Figure S18, Supporting Information). However, these nanoparticles likely resulted from the oxidation of desorbed Ir complexes in the electrolyte, rather than the direct decomposition of the adsorbed complexes. In operando Raman spectra confirmed the presence of the Ir complexes(ad) based on the C—N vibrational mode observed in dimethyl imidazole (Figure S19, Supporting Information), consistent with the XAS and XPS results (Figure 4 and S14, Supporting Information). We tested cyclic voltammetry with a bare ITO nanoparticle/FTO film in the dinuclear Ir complex dissolved electrolyte. Unlike the cyclic voltammetry (CV) result of Ir complex adsorbed ITO films in the nonaqueous electrolyte, an additional oxidation peak was found from the bare ITO film/FTO electrode in the Ir complex dissolved aqueous electrolyte (Figure S20, Supporting Information). Moreover, newly appeared IrO_*x*_ nanoparticles on ITO nanoparticles are confirmed from the TEM/EDS mapping image after the cyclic voltammetry test (Figure S21, Supporting Information). This result indicates that the mechanism of nanoparticle formation is because of the desorption and subsequent oxidation of Ir complexes (aq), rather than the direct oxidation of the Ir complexes(ad) onto ITO particles. Importantly, nanoparticle formation after stability tests was not observed for the thermally prepared IrO_*x*_ (Figure S22, Supporting Information).

### 2.2 In Operando Raman Spectroscopy

**Figure 5:**
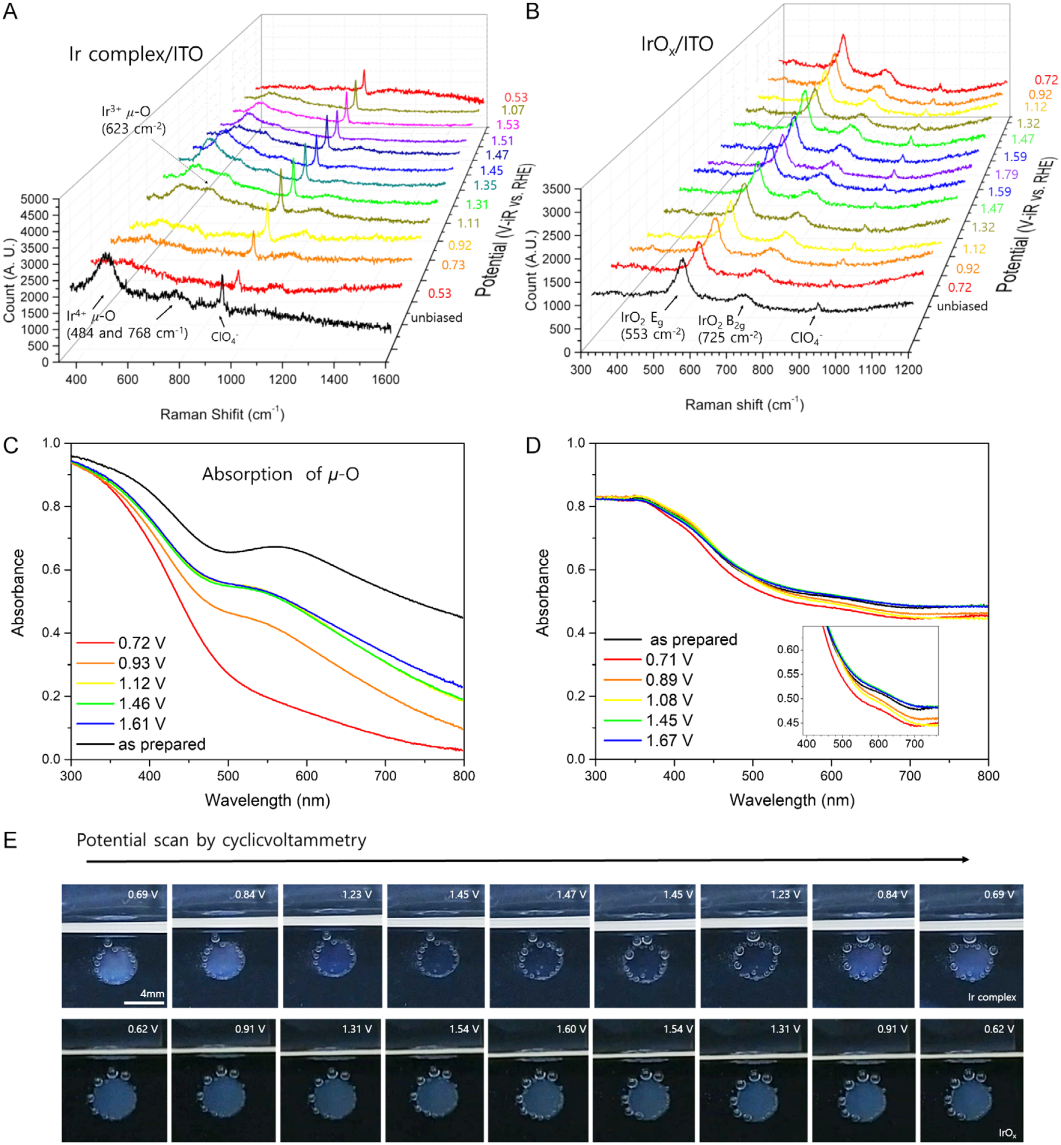
In operando Raman and UV–visible spectroscopy results of Ir complexes and IrO_*x*_. A,B) In operando Raman spectra of catalytic electrodes with adsorbed Ir complexes and IrO_*x*_ at different applied potential in 0.1 M KClO_4_ (pH 2). C,D) Ex situ UV–vis absorbance spectra of Ir complexes and IrO_*x*_‐adsorbed electrodes after applying constant potential. All potential scaleare versus RHE, and the potential values are iR compensated. E) A photograph of catalytic electrodes during cyclic voltammetry measurement. The oxygen bubbles near the active area originated from repeated cyclic voltammetry.

We investigated the state of the *μ*‐O bridges during OER by using in operando Raman spectroscopy (**Figure** **5**a). The Raman bands corresponding to Ir^4+^–*μ*‐O (484 cm^−1^), H_2_O–Ir^4+^–*μ*‐O (768 cm^−1^), and ClO_4_^−^ ions (933 cm^−1^) were identified in the Ir‐complex_(ad)_/ITO film under unbiased conditions, consistent with ex situ Raman spectroscopy^[^ ^13^, ^53^, ^54^ ^]^ (Figure S5a, Supporting Information). However, all of these *μ*‐O bands disappeared completely at relatively weak anodic potential (0.53–0.72*V*_RHE_), which was likely due to the reduction of Ir complexes changing the oxidation state of iridium from 4+ to 3+. In contrast, the similar analysis of IrO_*x*_ (Figure 5b) indicated that the bands did not shift nor disappear depending on the potential. The disappearance of the *μ*‐O bands was due to the cleavage of *μ*—O bonds, as confirmed by the UV–vis spectra (Figure 5c). The UV–vis absorption band at 586 nm, which indicates the presence of dinuclear Ir complexes, diminished at similar potential (0.53–0.72*V*_RHE_).^[^ ^55^ ^]^

Above ≈0.9*V*_RHE_, the Raman bands corresponding to Ir^4+^–*μ*‐O stretching (484 cm^−1^) and Ir^3+^–*μ*‐O (623 cm^−1^) reappeared at 1.11*V*_RHE_, and that corresponding to H_2_O–Ir^4+^–*μ*‐O (768 cm^−1^) reappeared at 1.35*V*_RHE_. The intensities of these bands continued to increase with increasing potential up to 1.4*V*_RHE_. This corresponds to the reformation of the Ir–*μ*‐O–Ir dinuclear structure from the two cleaved mononuclear Ir complexes with 3+/4+ mixed oxidation states. This was further supported by the UV–vis spectra (Figure 5c) showing increased absorbance at 586 nm at 0.9–1.4 *V*_RHE_. Notably, the complete disappearance and reappearance of Raman bands related to *μ*—O bonds represents unique behavior compared to IrO_*x*_/ITO (Figure 5b) and IrO_*x*_.^[^ ^55^ ^]^ This suggests that the cleaved *μ*—O bonds were completely restored to their original states, leading to high catalytic stability.

Above 1.4 *V*_RHE_, the Raman band corresponding to Ir^4+^–*μ*‐O stretching (484 cm^−1^) showed intensities decreased significantly. However, it is important to distinguish this behavior from the disappearance or weakening of the band at low potential (<0.92 *V*_RHE_). At high potential, the oxygen evolution from the Ir–*μ*‐O–Ir dinuclear structure, which may involve the cleavage of the *μ*—O bonds, is activated along with the *μ*‐O restoration reaction. This results in an equilibrium between *μ*‐O cleavage and restoration, as reflected by the decreased intensities of the *μ*‐O bands in the Raman spectra.

We also found that the band intensity of Ir^4+^–*μ*‐O decreased above 1.4 *V*_RHE_, while that of Ir^3+^–*μ*‐O remained relatively constant. This corresponds with the increased and saturated absorbance at 586 nm at 1.46 and 1.61 *V*_RHE_ (Figure 5c), and the color change observed in the Ir complex‐immobilized electrode during cyclic voltammetry at different potential (Figure 5e). The Ir complex‐immobilized catalytic electrode was initially indigo blue due to strong absorption at 586 nm, but became pale blue after the cleavage of *μ*—O bonds at low applied potential. At high applied potential, we observed the formation of oxygen bubbles, and the color reverted to the indigo blue, even though the intensity of Raman bands was slightly decreased at 1.47 *V*_RHE_. However, we did not observe the restoration of *μ*—O bonds in IrO_*x*_, which was likely related to the lower activity and stability of IrO_*x*_/ITO compared to our Ir complexes in the OER. In contrast, the Raman spectra of IrO_*x*_, obtained under varying potential, show only two peaks at 553 and 725 cm^−1^, corresponding to IrO_2_ *E*g and B2g peaks.^[^ ^56^ ^]^ Despite the confirmed change in the oxidation state of Ir at ≈1.05 *V*_RHE_ (Figure S23, Supporting Information), there are no significant peak shifts or the appearance of new peaks. UV–vis spectra of an IrO_*x*_/ITO film at the different potential show consistent absorption characteristics (Figure 5d). As a result, no indications of *μ*—O bond regeneration from IrO_x_ were found. The constant intensities of the *E*_g_ and B_2g_ bands suggest that IrO_*x*_ follows an irreversible LOM for OER. This irreversible OER mechanism may explain the relatively lower performance of IrO_*x*_/ITO compared to our Ir complexes in OER.

Lastly, the shifting of the Raman bands for *μ*—O bonds of Ir complexes in a 0.1 M KClO_4_ H_2_^18^O electrolyte supported the potential involvement of oxygen atoms in the *μ—*O bonds in the release of oxygen gas during OER. It has also been reported that the superoxide motif in mono‐ or *μ*‐O bridges is released as O_2_, and the *μ*–O bridge is regenerated from oxygen atoms of water molecules during OER through LOM. During the OER reaction in H_2_^18^O electrolytes, the Raman band of *μ*‐^16^O of our Ir complex catalyst is red‐shifted due to the formation of newly *μ*‐^18^O in the KClO_4_ H_2_^18^O electrolyte (Figure S24, Supporting Information). A similar shift in Raman band behavior has been reported in the case of Co‐*μ*‐O ‐Co of CoOOH catalysts in K^18^OH electrolytes.^[^ ^57^ ^]^ It is important to note that the *μ*–O cleavage/regeneration process of Ir complexes is reversible; all *μ*‐O peaks disappear when potential is applied backward (from high to low).

### 2.3 Theoretical Analysis

DFT calculations were employed to explain the enhanced OER activity of the Ir complexes anchored on ITO (referred to as the anchoring effect), and the appearance of *μ*‐O peaks in the Raman spectra at 1.1–1.6 *V*_RHE_, but not lower than 1 *V*_RHE_.

**Figure 6:**
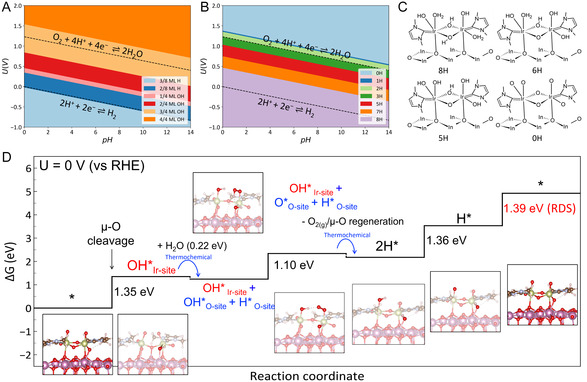
DFT calculation results of Ir complexes. A) Calculated surface Pourbaix diagram for the iridium tin oxide (ITO) (111) surface. The black dashed lines indicate the equilibria of OER (1.23 V vs RHE) and HER (0 V vs RHE). The legend represents the adsorbate's surface coverage. B) Number of protons remaining on the Ir complex anchored on the ITO surface under different conditions (applied potential and pH). The two dashed lines indicate the equilibria of OER and HER. C) Schematic drawings of the Ir complexes anchored on the ITO surface presented in B). D) Free energy diagram for OER on the fully deprotonated Ir complex (no protons remaining) anchored on the ITO (111) surface at U = 0 V. The optimized structures for each reaction step are shown. The value of the reaction‐free energy for the rate‐determining step is shown in red. The blue arrows show that the reaction step is a thermochemical process, and the calculated free energy barrier of the thermochemical water dissociation is 0.22 eV.

First, the fully protonated (unanchored) Ir complex was determined to retain approximately four protons at 1.2–1.8 *V*_RHE_ (Figure S25, Supporting Information), resulting in the relatively high limiting potential of 2.14 *V*_RHE_ (Figure S26, Supporting Information). The anchoring of this Ir complex to an ITO support can manifest through two distinct reactions, contingent upon the pH‐associated surface coverage. At pH < 7, the reaction involves [Ir]−2OH + 2 H* O‐site → [Ir]*O‐site + 2H_2_O, whereas, at pH > 7, it proceeds via [Ir]−2OH + 2OH* In‐site → [Ir]−2O* In‐site + 2H_2_O (**Figure** **6**a and S27, Supporting Information). The former reaction was found to be 0.84 eV more favorable, consistent with the more efficient binding of Ir complexes to ITO supports under acidic conditions (further details in the theoretical section of Supporting Information).

In contrast, we found that complete deprotonation of the Ir complex anchored on ITO is favorable at the reduced potential of 1.45 *V*_RHE_ (Figure 6b), in contrast to the unanchored case. The free energy diagram for OER on the anchored Ir complex (Figure 6d) showed a significant reduction in the reaction free energy for the OH* formation step (2.14 → 1.35 eV) due to the close interaction with the ITO surface. This interaction facilitated the direct formation of OH* on Ir, bypassing the involvement of dangling oxygen. Furthermore, the *μ—*O bonds underwent 0.2 Å elongation (Figure S28, Supporting Information) when the Ir complex was supported on the ITO surface, facilitating OH* formation on the Ir site.

Three reaction routes become feasible after OH* formation on the Ir site of the anchored Ir complex: 1) electrochemical oxidation of the OH* to O*; 2) additional OH* formation via electrochemical water dissociation; and 3) thermochemical water dissociation (H_2_O → OH* O‐site + H* O‐site). The former two routes were highly endothermic by 1.73 and 1.56 eV (Figure S29, Supporting Information). Conversely, the third route was slightly exothermic with the low reaction free energy of –0.11 eV and the small free‐energy barrier of 0.22 eV (Figure 6d). Note that the third route became feasible only upon anchoring the Ir complex on the ITO surface. For thermochemical water dissociation after OH* formation (OH* Ir‐site + H_2_O → OH* Ir‐site + OH*O‐site + H* O‐site), two conditions must be met: 1) the *μ*—O bond should be easily breakable to create two stabilized iridium sites hosting OH* species (OH* Ir‐site and H* O‐site, equivalent to OH* Ir‐site); and 2) the Ir complex should be fully deprotonated to enhance the reactivity of the dangling oxygen site where OH* O‐site can be stabilized.

**Figure 7:**
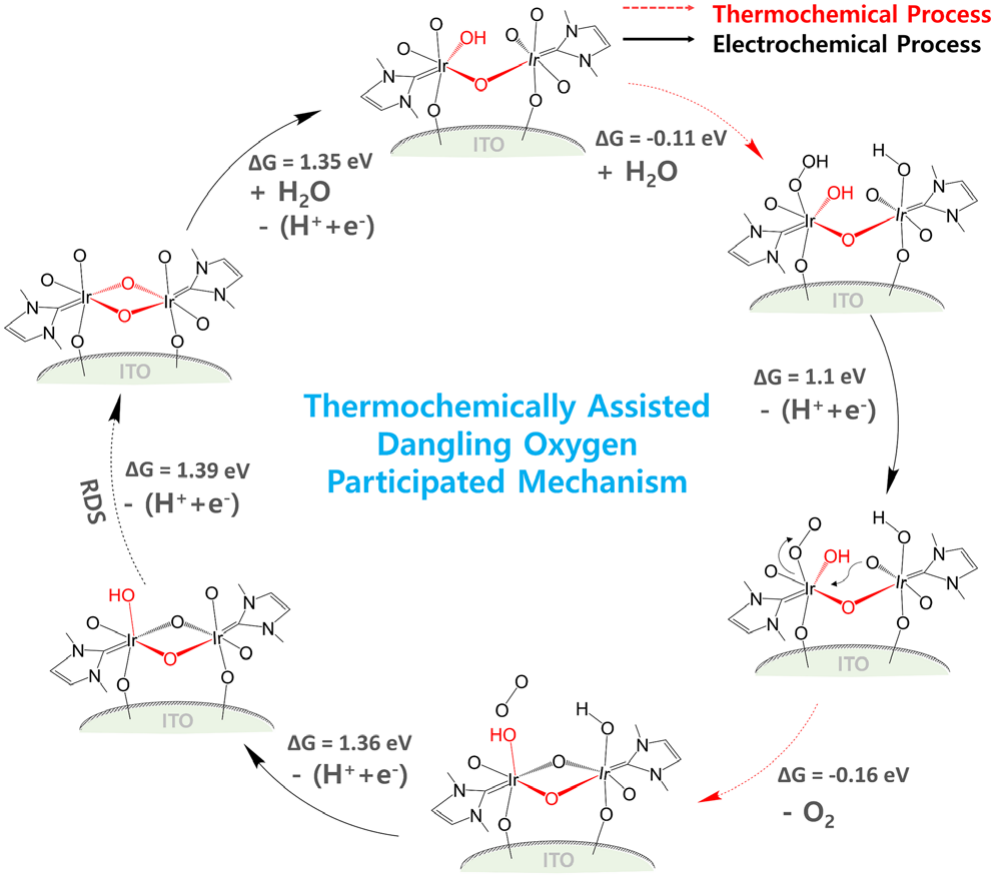
t‐DOM mechanism of Ir complexes during OER. The schematic diagram of the thermochemically assisted dangling oxygen participation mechanism (t‐DOM) in the immobilized dinuclear Ir complex for OER based on DFT calculations.

Following thermochemical water dissociation on the Ir complex, the OER proceeds similar to the LOM in highly reducible perovskites.^[^ ^58^ ^]^ OH* O‐site deprotonates to form OO‐site and desorbs as O_2_(g), creating an oxygen vacancy (OV) (OH* Ir‐site + O * O‐site + H* O‐site → OHIr‐site + O_2_ + OV + HO‐site). The Ir‐complex promptly restructures to fill the vacancy, restoring the *μ—*O bond and resulting in the 2H* O‐site state (OH* Ir‐site + OV + H* O‐site → 2H* O‐site). The complex reverts to its original state by deprotonating two protons with the reaction‐free energy of 1.39 eV, closely matching the experimentally obtained onset potential of 1.41 *V* − *iR*. This unique thermochemical water dissociation event during the OER significantly reduced the limiting potential from 2.14 to 1.39 *V*_RHE_, which is attributed to the thermochemically assisted dangling oxygen participation mechanism (t‐DOM, **Figure** **7**).

The theoretically identified t‐DOM in the anchored Ir complex aligns with potential‐dependent Raman spectra (Figure 5). Under unbiased conditions, *μ*‐O peaks (at ≈484 and 768 cm^−1^) indicated full protonation of the Ir complex on the H*‐covered ITO surface, totaling eight attached protons ([Ir]–8H*). These peaks vanished at 0.5–1 *V*_RHE_ as OH* covers the ITO surface, leaving only six protons on the Ir complex ([Ir]–6H*). The OH* interaction with [Ir]–6H* formed [Ir]–O–7H* with the low free‐energy barrier of 0.17 eV, causing *μ—*O bond breakage (Figure S30a, Supporting Information). Because [Ir]–O–7H* remained highly protonated, thermochemical water dissociation cannot occur and the *μ*—O bonds are not regenerated via the OER, consistent with the absence of the *μ*‐O peaks at 0.5–1 *V*_RHE_.

The *μ*‐O peaks reappeared above 1.0 *V*_RHE_, peaking at 1.35 *V*_RHE_ due to high oxidation potential causing severe deprotonation of [Ir]–O–7H* to [Ir]–O–1H* (Figure S30b, Supporting Information), which is identical to the second reaction state (OH* Ir‐site) in Figure 6d. Further oxidation to O_2_(g) + 2H* can occur below 1.1 *V*_RHE_, but the complete OER via t‐DOM requires 1.39 *V*_RHE_. This means that, at potentials lower than 1.39*V*_RHE_, the reaction stops at either the H* O‐site or 2H* *O*‐site stage, after fully regenerating the *μ—*O bonds (Figure S29c, Supporting Information). This accounts for the reappearance of *μ*‐O peaks at potentials above 1.0 *V*_RHE_ and their maximum intensities just below 1.39 *V*_RHE_.

Finally, the Raman spectra (Figure 5) showed that the *μ*‐O peaks decreased by half as the potential increased above the limiting potential for OER via t‐DOM (1.39 *V*_RHE_). This occurred because, at higher oxidation potential, the thermodynamics allow for both the cleavage and regeneration of the *μ*—O bonds, thereby reducing the chance of detecting these bonds through Raman spectroscopy by approximately half compared to that at the maximum intensities.

## 3 Conclusion

We immobilized highly active and stable dinuclear Ir complexes on ITO supports for electrochemical OER in acidic media. The slightly distorted and elongated *μ—*O bonds of the immobilized Ir complex were easily cleaved, thereby generating dangling oxygens that promote thermochemical water dissociation at room temperature. Consequently, oxygen gas was released at lower potential via the participation of the dangling oxygens while regenerating the *μ—*O bonds. This t‐DOM, likely specific to immobilized molecular catalysts, resembles the widely accepted LOM for relatively unstable (highly reducible) metal oxides but has the advantage of higher catalytic stability in acidic media. The new mechanism described in this study provides an empirical basis for the design of immobilized molecular catalysts with enhanced performance.

## 4 Experimental Section

4.1

4.1.1

##### Synthesis of Complex 1, Dichloro(pentamethylcyclopentadienyl)(1,3‐dimethylimidazol)Iridium

Silver oxides (0.175 g, 0.75 mmol) were added to a solution of 1,3‐Dimethylimidazollium iodide (TCI, 98.0%) (0.281 g, 1.26 mmol) in CH_2_Cl_2_ (10 mL) in the dark. The solution was stirred at the room temperature for 2 h, and [Cp*IrCl_2_]_2_ (Pentamethylcyclopentadienyl iridium chloride dimer (Aladine, 96%)) (0.500 g, 0.63 mmol) was added to the solution. The mixture was stirred at the room temperature for 1 h and filtered through Celite. The solvent was evaporated and the crude solid was dissolved in CH_2_Cl_2_/methanol and purified by a SiO_2_ column chromatograph. Elution with CH_2_Cl_2_/methanol (15:1) and then CH_2_Cl_2_/methanol (10:1) yielded the orange crystals of 1 (0.479 g, 0.97 mmol, 77%).

^1^H NMR (600 MHz, CDCl_3_): 6.90(d, 22H, H_im_), 3.94(s, 6H, CH_3_), 1.60(s, 15H, Cp*).

##### Synthesis of Complex 2, Dihydroxyl(pentamethylcyclopentadienyl)(1,3‐dimethylimidazol)Iridium

The complex **1** (0.284 g, 0.57 mmol) was added to a mixture of Ag_2_O (Sigma‐Aldrich, 99.0%) (0.160 g, 0.69 mmol) in H_2_O (15 mL) in the dark. After a couple of minutes, the complex became soluble. The mixture was stirred for 4 h and filtered through Celite. The solvent was evaporated and then the mixture was re‐dissolved in CH_2_Cl_2_. It was evaporated again to remove water more.

^1^H NMR (600 MHz, CD_2_Cl_2_): 6.96(d, 2H, H_im_), 3.80(s, 6H, CH_3_), 1.75(s, 15H, Cp*).

##### Synthesis of 3, [Ir(dmimd)(OH)(H_*2*_ *O)*_*2*_ *(μ‐O)]*_*2*_^*2+*^

**Scheme 1:**
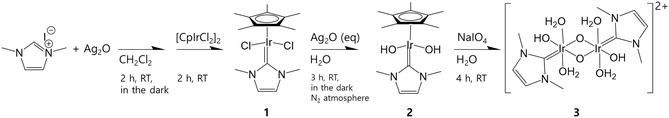
A schematic diagram for synthesis of iridium molecular complexes.

The complex **2** (0.129 g, 0.28 mmol) was added to a mixture of NaIO_4_ (1.2 g, 5.64 mmol) in H_2_O (25 mL) in an ice bath. The solution was stirred at the room temperature for 4 h and a visibly blue color slowly appeared while stirring. The dimerization of complex **2** was confirmed by ^13^C NMR peak shifting (**Scheme** **1**).

^13^C NMR of complex **2** (600 MHz, D_2_O): *δ* 123.4, 86.1, 36.6, 8.5.

^13^C NMR of complex **3** (800 MHz, D_2_O): *δ* 177.4, 159.1, 24.6, 20.0.

##### Support and Immobilization of Catalysts on the Conducting Substrate

A conductive mesoporous layer of In_2_O_3_ · SnO_2_ (ITO) nanoparticles was deposited on F‐doped SnO_2_‐glass substrates (FTO) by a doctor‐blade method with ITO pastes. To prepare the ITO paste, ITO nanoparticles (nanopowder, <50 nm particle size, Sigma‐Aldrich), ethylcellulose (48.0–49.5% (w/w) ethoxyl basis, Sigma‐Aldrich), and terpineol (the mixture of isomers, anhydrous, Sigma‐Aldrich) were dispersed in an ethanol solvent with ultra‐sonication for 1 h. The weight ratio of the mixture was 1:0.4:4. Subsequently, the ethanol was removed from the mixture by a rotary evaporator, then the ITO paste was obtained. After a doctor‐blade method, as‐deposited substrates were annealed at 500 °C in the air atmosphere for 1 h. The thickness of ITO layers was controlled by using a spacer during the doctor‐blade method. The final thickness was 8 μm with 2 layers of the spacer (3 M Scotch Magic Tape 810, 3 M). Ir complex molecules were adsorbed on mesoporous ITO layers by a dipping method. A substrate was dipped in a 1 mM [Ir(dmimd)(OH)(H_2_O)(*μ‐*O)_2_]^2+^ aqueous solution for 12 h. The solution was kept in a dark room. The ITO layer with adsorbed Ir complexes was rinsed with and dried by a nitrogen blower. The color of films changed to deep blue after the chemisorption of Ir complexes. It was confirmed by UV–vis spectroscopy. The [Ir(dmimd)(OH)(H_2_O)(*μ‐*O)_2_]^2+^ show specific absorption at 586 nm (Figure S31a, Supporting Information), and the Ir complexes adsorbed film also shows the same absorption peak at 586 nm (Figure S31b, Supporting Information) To prepare IrO_*x*_/ITO films, Ir complex adsorbed ITO films were annealed in the air atmosphere at 500 °C for 1 h with a 5 °C min^−1^ ramping rate. The Ir complex is converted to IrO_*x*_ by thermolysis, then the absorption peak is shifted to 610 nm in the UV–vis spectroscopy. (Figure S31c, Supporting Information).

##### Electrochemical Characterization

All electrochemical characterization was carried out by a potentiostat (PGSTAT128N, Metrohm) with a conventional three‐electrode system (Ag/AgCl reference electrode (3 M NaCl), a platinum counter electrode, and working electrodes (catalytic electrodes with adsorbed Ir complexes)). The active area of the working electrode was controlled as 0.1256 cm^2^. I–V curve was obtained to investigate the properties of catalytic electrodes for OER by cyclic voltammetry (scan rate, 10 mV sec^−1^) in a 0.1 M KClO_4_ (pH 1–5), 0.1 M KNO_3_ (pH 2), 0.1 M K‐Pi buffer (pH 7), 0.5 M Na_2_SO_4_ (pH 5.43) solution or a 0.1 M phosphate‐citrate buffer solution (pH 3.66). The pH of KClO_4_ and KNO_3_ solution was adjusted by a 0.1 M HClO_4_ solution. All chemicals used for preparing the electrolyte were obtained from Sigma‐Aldrich. The potential scale versus an Ag/AgCl electrode was converted to a reversible hydrogen electrode (RHE) by Equation (1)

(1) $$V_{\text{RHE}}=V_{\text{Ag}/\text{AgCl}}+(\text{pH}\times 0.0591)+V_{\text{Ag/AgCl}}^{0}\;(3M\;\text{NaCl})$$

where *V*_RHE_ is the potential versus RHE, *V*_Ag/AgCl_ is the applied potential versus the Ag/AgCl electrode, $V_{\text{Ag}/\text{AgCl }}^{0}$is the standard potential of the Ag/AglCl electrode, 0.209 V. The solution resistance between the reference electrode and the working electrode in various electrolytes was compensated with *I*–*V* results by Equation (2) below

(2) $$V_{\text{compensated}}\left(V\right)=V_{\text{measured}}\left(V\right)-\left|I\right|(A)\times R_{\text{solution}}(\Omega )$$

where *V*_compensated_ is the compensated potential, *V*_measured_ is the applied potential during the cyclic voltammetry, *R*_solution_ is the solution resistance between a working electrode and the reference electrode. The compensated potential in this article is always noted as “*V*‐*iR*,” otherwise not the compensated potential. The *R*_solution_ of a three‐electrode system was estimated by electrochemical impedance spectroscopy (EIS) under the same condition after every cyclic voltammetry measurement. The detail measurement condition of EIS was the frequency range from 5000–1 Hz at 1 V versus Ag/AgCl applied sigmoidal potential. To estimate the Tafel slope of catalytic electrodes, chronoamperometry was carried out for 30 s for each 0.9–1.6 V versus Ag/AgCl potential. The average current density collected from the chronoamperometry at each potential was plotted as overpotential (V) versus log (J). For a stability test, chronoamperometry was carried out for 12 or 16 h in the H‐cell which has a cathode and an anode separated by a proton exchange membrane (Nafion 117). The constant potential was applied to electrodes for obtaining the current density of 10 mA cm^−1^ in the different electrolytes.

The evolved amounts of oxygen gas during electrolysis were measured by a gas chromatograph (YL6500 GC, YOUNGIN Chromass). A molecular sieve 5A column and a plasma discharge ionization detector (PDD) were used. Faradaic efficiency was calculated from the coulomb passed during the electrolysis and O_2_ gas accumulated in the electrochemical reactor by Equation (3) and (4) below, where *ϕ* is the oxygen volumetric concentration, *Q* is the flow rate of feed gas (He, 99.9999%) which is injected to the GC (20 mL min^−1^), *n* is the equivalent electron number (4, 2H_2_O → O_2_ + 4H^+^ + 4*e* ^−^), *F* is the Faraday constant (96485.3329 C mol^−1^), *p* is the pressure of the electrochemical cell (1 atm), *R* is the ideal gas constant (0.082057 L atm K^−1^ mol^−1^), *T* is the reactor temperature (298 K) of the electrochemical cell, *I*_*ϕ*_ is the current which is calculated from the evolved gas amount, *I*_stat_ is steady‐state current during electrolysis.

(3) $$\phi \times Q\times \frac{nFp}{RT}=I_{\phi }$$

(4) $$\frac{I_{\phi }}{I_{\text{Stat}}}\times 100\%=\eta_{F}$$

##### TOF Calculation

The TOF of catalytic electrodes was calculated by Equation (5) below

(5) $$\text{TOF}=\frac{\text{rate}\;\text{of}\;\text{oxygen}\;\text{evolution}\;\text{reaction}}{\text{number}\;\text{of}\;\text{catalytic}\;\text{active}\;\text{site}}=\frac{\text{Current}\;\text{density}\left(A\;{\text{cmL}}^{-2}\right)/n\cdot F\left(s\;A\;{\text{mol}}^{-1}\right)}{0.392\;\pm \;0.074\left(\text{μmol}\;{\text{cmL}}^{-2}\right)}$$

where *n* is the equivalent number of the electron for the water oxidation, and *F* is the Faraday constant. In this research, we use the geometrical current density for the reaction rates because our electrocatalyst is a film type. However, the number of active sites should be considered on the actual surface area to avoid underestimating the active site. We use two assumptions, the first is that every Ir molecular complex in the active area contributes to the water oxidation, and the second is that one Ir molecular complex ([Ir(dmimd)(OH)(H_2_O)_2_(*μ*‐O)]_2_^2+^) has two active sites (*μ*‐O). With these assumptions, the half of the Ir complex adsorbed is equal to the number of the active site.

The concentration of Ir complexes adsorbed was calculated by UV–vis spectroscopy. A [Ir(dmimd)(OH)(H_2_O)_2_(*μ*‐O)]_2_^2+^ aqueous solution has an absorption peak at 586 nm wavelength, so a calibration curve for the peak area versus concentration was obtained by a plastic cuvette (1 cm) and an UV–vis–near‐infrared spectrometer (Cary 5000, Agilent) as Figure S32, Supporting Information.

##### In Operando Raman Spectroscopy

Raman spectroscopy was measured in a Via confocal Raman Microscope (Renishaw) with a 532 nm laser or a 785 nm laser regardless of ex situ or in operando measurement. For the in operando measurement, we designed a 3 electrode electrochemical cell (Figure S33, Supporting Information). The cell mounted inside the spectrometer was connected to a potentiostat (Iviumstat, Ivium) applying potential. Electrolytes are exposed to the atmosphere to improve the signal intensity of the spectrum and avoid accumulating the oxygen gas which repels electrolytes from the electrode. Significantly, our electrochemical cell facilitated the execution of in operando Raman spectroscopy at a comparable reaction rate condition (>10 mA cm^−2^). The steady‐state current‐potential plot presented aligns well with the outcomes of the CV (Figure S34, Supporting Information and Figure 3c). The added advantage of our system is that it does not require specialized substrates, such as gold or other metal substrates typically utilized for Raman signal amplification via surface plasmon effects. Instead, the same electrode is simply mounted onto our custom‐designed cell.

##### Other Characterization

The chemical structure of Ir complexes or adsorbed Ir complexes on ITO nanoparticle layer/FTO glass substrate was investigated by Fourier transformed nuclear magnetic resonance spectroscopy ((FT‐NMR), (Avance 400, Bruker), (DD2 600, Agilent), (800/45 ASCENDTM, Bruker)), FT‐IR (Nicolet iS10, Thermo Fisher), XPS (PHI 5000 VersaProbe, Ulvac‐PHI), and XAS 8C beam‐line of the pohang accelerator laboratory (PAL). All XAS signal were obtained by florescence mode due to presence of FTO‐coated glass substrates. HR‐TEM and EDS elemental mapping were carried out by a transmission electron microscope (TitanTM 80‐300 and Talos F200X, FEI). In FTIR analysis, any signals related to the Ir complex were not revealed after immobilization, however, ex situ Raman spectroscopy detected two peaks indicating the presence of *μ*–O bridges (484 and 768 cm^−1^) and the C—N vibrational mode in dimethyl imidazole (1360 cm^−1^) (Figure S5, Supporting Information). HR‐TEM could not be used to examine the Ir complex immobilized on the ITO lattice due to decomposition caused by the incident electron beam (Figure S6, Supporting Information). Similar observations of metal complex decomposition under electron beam exposure have been reported previously.^[^ ^21^, ^22^ ^]^

##### Theoretical Analysis of [Ir(dmimd)(OH)(H_*2*_ *O)*_*2*_ *(μ‐O)]*_*2*_^*2+*^

We have first determined the degree of deprotonation of unanchored Ir complexes under anodic conditions. Figure S25, Supporting Information, shows that, at the equilibrium potential of 1.23 *V*_RHE_, the fully protonated Ir complex loses six protons while retaining four. Deprotonating the seventh proton requires over 2 *V*_RHE_. Figure S26, Supporting Information, shows the free energy diagram for OER on the Ir complex with four protons attached (namely, Ir complex−4H). The reaction favors proceeding via the participation of dangling oxygens compared to the adsorbate‐evolving pathway. However, both pathways are still limited by the precedent step of extremely uphill OH* formation (H_2_O à OH* + H^+^ + *e* ^−^), and require relatively high limiting potential of 2.14*V*_RHE_.

##### Theoretical Analysis of Anchoring of Ir complexes

We have then investigated the anchoring reaction of Ir complexes on ITO supports. Figure 6a shows the surface Pourbaix diagram for pristine ITO (111). At pH < 7, the ITO surface is covered with H* that binds to oxygen sites. This means that the Ir complex anchors on the surface via [Ir]−2OH + 2 H*_O‐site_ → [Ir]*_O‐site_ +2H_2_O resulting in a chemical bond between iridium of the Ir complex and lattice oxygens of ITO (Figure 7a). In contrast, at pH > 7, the ITO surface is covered with OH* that binds on indium sites. This means that the Ir complex anchors on the surface via [Ir]−2OH + 2OH*_In‐site _→ [Ir] −2O*_In‐site_ + 2H_2_O resulting in a chemical bond between the dangling oxygen of Ir complexes and indium of ITO (Figure 7b). We find that the former anchoring reaction is 0.84 eV more favorable than the latter. This is in agreement with experiments that Ir complexes bind on ITO supports only under acidic conditions, and the catalytic performance degrades significantly as pH increases.

##### Computational Methods

DFT calculations for (unanchored) Ir molecular catalysts were performed using the ORCA 5.0.2 program package.^[^ ^59^ ^]^ The PBE functional^[^ ^60^ ^]^ was employed in conjunction with the def2‐TZVP basis set^[^ ^61^ ^]^ The conductor‐like‐polarizable continuum model (CPCM)^[^ ^62^ ^]^ was used to account for the water solvent effect.

For Ir complexes anchored on the Indium tin oxide (ITO) surface, the Vienna Ab initio Simulation Package (VASP) 5.4.4^[^ ^63^ ^]^ was utilized. The PBE‐D3 functional^[^ ^60^, ^64^, ^65^ ^]^ and projector augmented wave method^[^ ^66^ ^]^ were used. The energy cutoff for all calculations was set at 500 eV. We used a (4 × 4 × 4) and (2 × 2 × 1) Monkhorst–Pack k‐point mesh^[^ ^67^ ^]^ for bulk and surface calculations, respectively. To reduce computational costs, we anchored Ir complexes on In_2_O_3_ (111) instead of ITO (111). This is because the atomic % of tin in ITO is small (≈3.6%),^[^ ^68^ ^]^ requiring a large slab model. In previous studies, (111) has been shown to be the most stable facet among low‐index surfaces of In_2_O_3_.^[^ ^69^ ^]^ To account for the effect of tin on the ITO surface Pourbaix diagram, we substituted a few indium atoms with tin, resulting in a slightly higher atomic % of tin than that of ITO. All structural relaxations were conducted until the energy difference was less than 10^−5^ eV and the force was less than 0.03 eV Å^−1^. The activation barrier was calculated using the Nudged Elastic Band method.^[^ ^70^ ^]^

## Conflict of Interest

The authors declare no conflict of interest.

## Author Contributions

**Sang Youn Chae**: conceptualization (lead); data curation (lead); investigation (lead); methodology (lead); project administration (supporting); visualization (lead); writing—original draft (lead); writing—review & editing (lead). **Myeong Jin Choi**: data curation (lead); formal analysis (lead); investigation (lead); methodology (lead); visualization (lead); writing—original draft (lead). **Si Young Lee**: formal analysis (equal); investigation (equal); methodology (equal). **Ja Yoon Choi**: (formal analysis (equal); investigation (equal); writing—original draft (equal). **Dae Won Kim**: (formal analysis (equal); investigation (equal). **Je Seung Lee**: investigation (equal); methodology (equal); writing—original draft (equal). **Eun Duck Park**: funding acquisition (equal); project administration (equal); supervision (equal); validation (equal); writing—review & editing (equal). **Jong Suk Yoo**: data curation (lead); formal analysis (equal); validation (equal); visualization (equal); writing—original draft (equal). **Oh‐Shim Joo**: conceptualization (lead); funding acquisition (equal); project administration (lead); supervision (lead); writing—review & editing (lead). **Sang Youn Chae** and **Myeong Jin Choi** contributed equally to this work.

## Supporting information

Supplementary Material

## Data Availability

The data that support the findings of this study are available from the corresponding author upon reasonable request.
