# Supplementary material for: Highly Active and Stable Immobilized Iridium Complexes via Thermochemically Assisted Dangling Oxygen Participation for Electrochemical Oxygen Evolution Reaction
Source: Small Sci. 2025 May 9;5(7):2500027. doi: 10.1002/smsc.202500027 (PMC12257893; doi:10.1002/smsc.202500027)
Supplement: Supplementary file 1 — Supplementary Material [file SMSC-5-2500027-s001.pdf]

Supplementary Information for

Highly Active and Stable Immobilized Iridium Complexes via Thermochemically Assisted Dangling Oxygen Participation for Electrochemical Oxygen Evolution Reaction

Sang Youn Chae,[1,2,†] Myeong Jin Choi,[3,†] Si Young Lee,[4] Ja Yoon Choi,[5] Dae Won Kim,[6] Je Seung Lee,[6] Eun Duck Park,[1,7] Jong Suk Yoo,[3,*] and Oh-Shim Joo[5,**]

[1]Department of Energy Systems Research, Ajou University, Suwon 16499, Republic of Korea
[2]Ajou Energy Science Research Cneter, Ajou University, Suwon 16499, Republic of Korea
[3]Department of Chemical Engineering, University of Seoul, Seoul 02504, Republic of Korea
[4]Department of Mechanical Engineering, University of Michigan, Ann Arbor, Michigan 48109, United States, United states of America
[5]Clean Energy Research center, Korea Institute of Science and Technology, Seoul 02792, Republic of Korea
[6]Department of Chemistry, Kyung Hee University, 02447 Seoul, Republic of Korea
[7]Department of Chemical Engineering, Ajou University, Suwon 16499, Republic of Korea

†These authors contributed equally
*Correspondence: jsyoo84@uos.co.kr
**Correspondence: joocat@kist.re.kr

Supplementary Figures

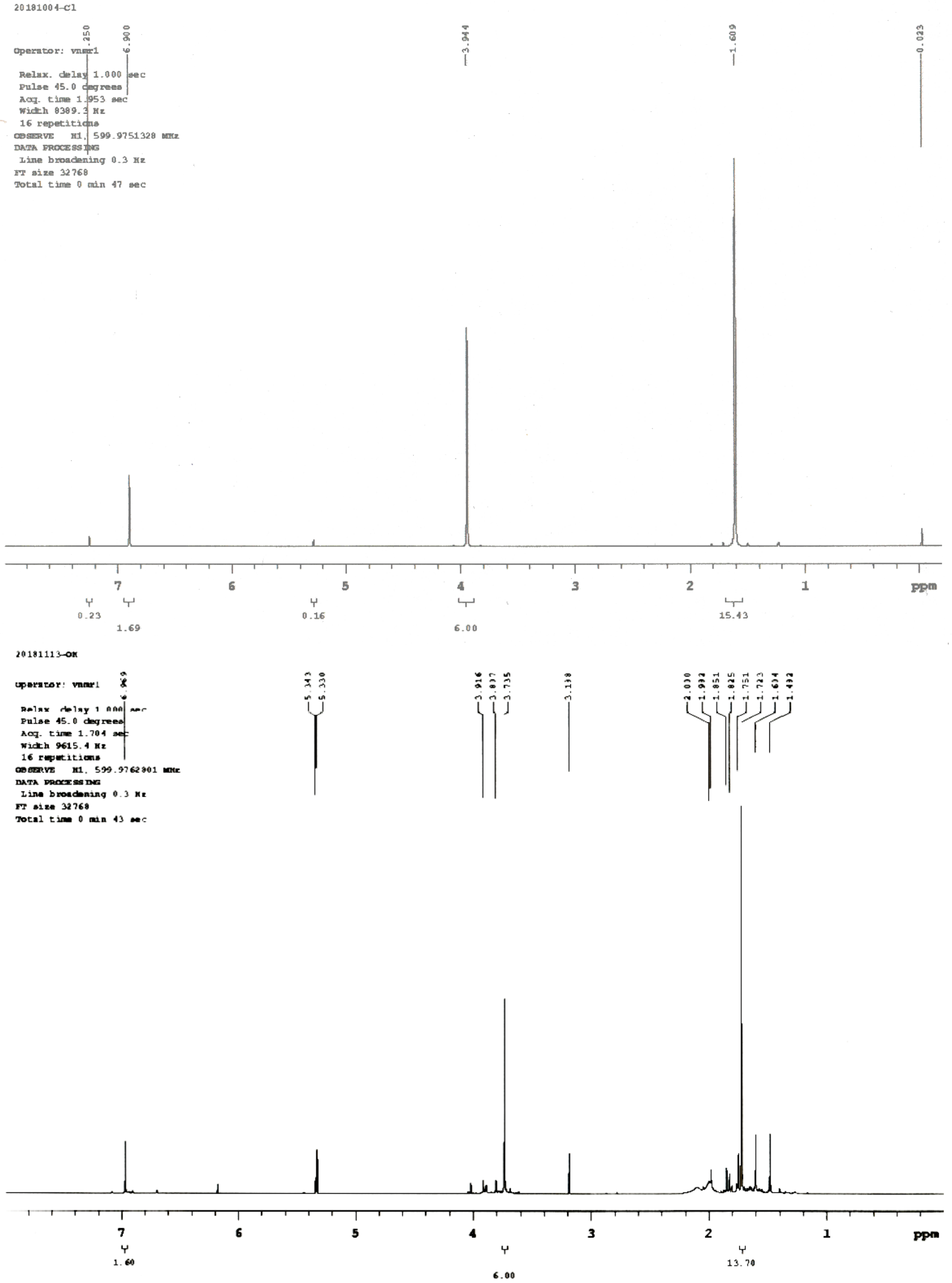

Figure S1. ^{1}H NMR spectra of synthesized complex **1** (top) and **2** (bottom)

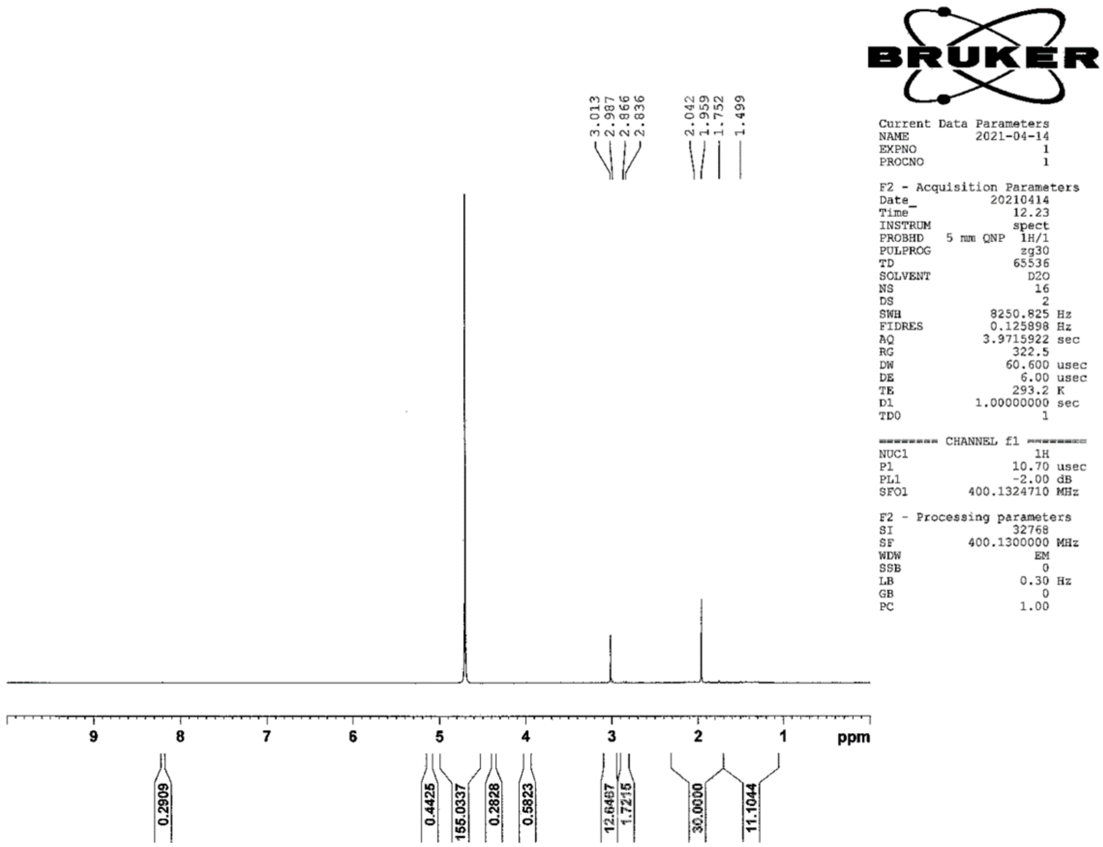

Figure S2. ^{1}H NMR spectrum of synthesized complex **3**

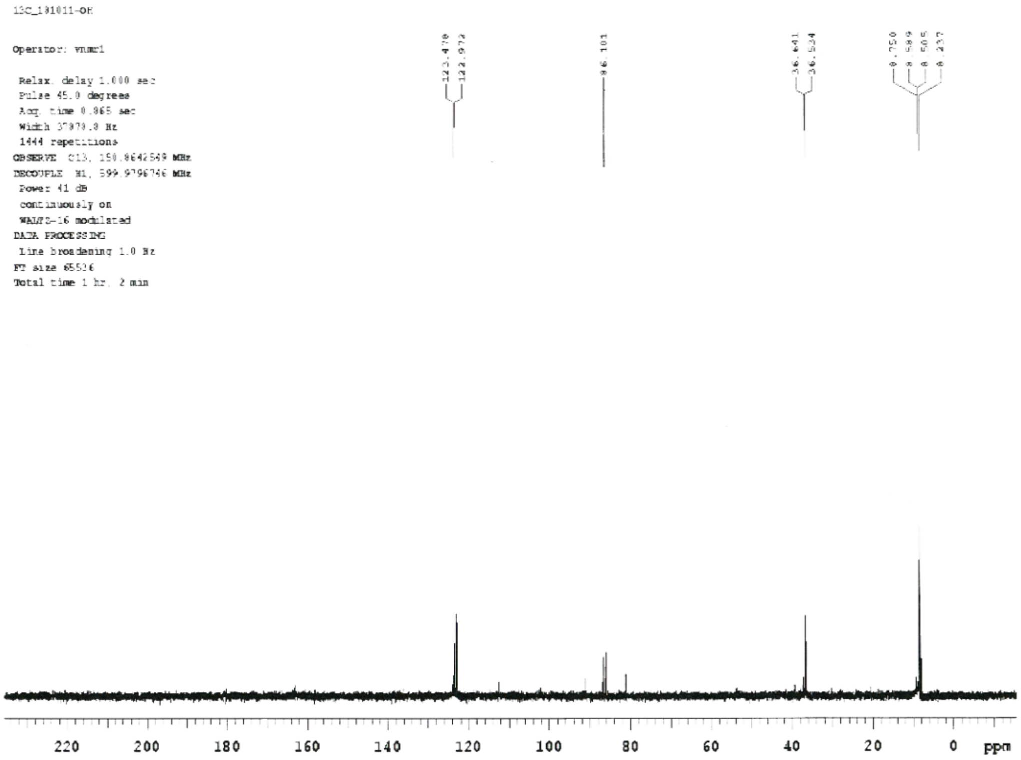

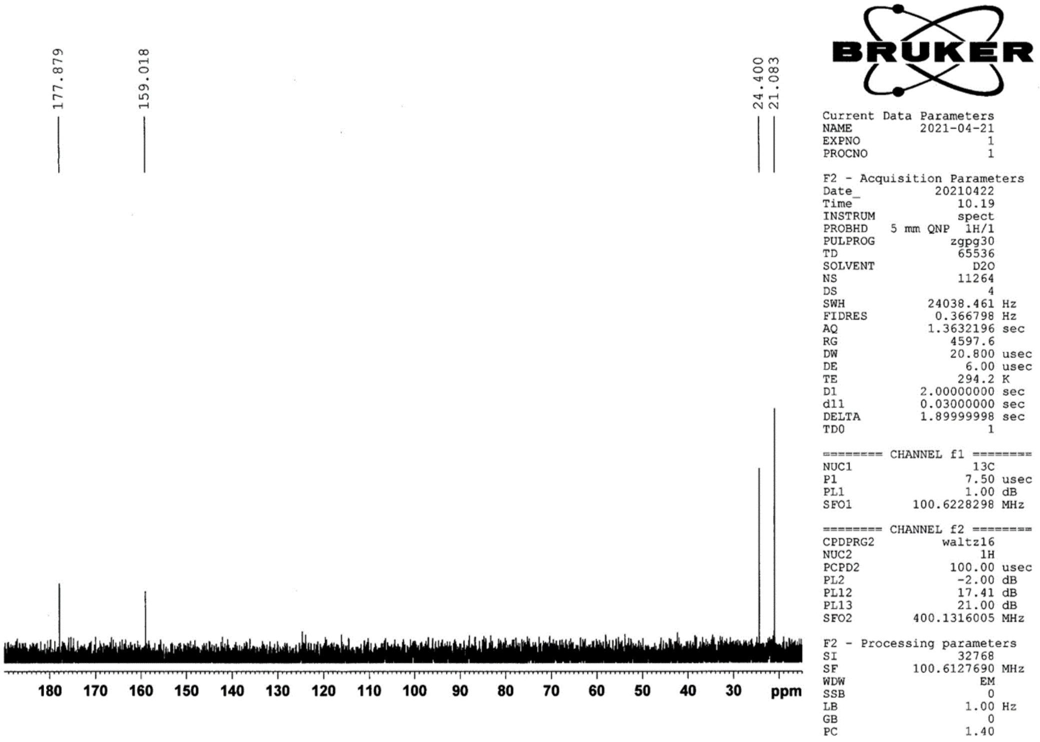

Figure S3 ^{13}C NMR spectra of synthesized complex **2** (top) and **3** (bottom)

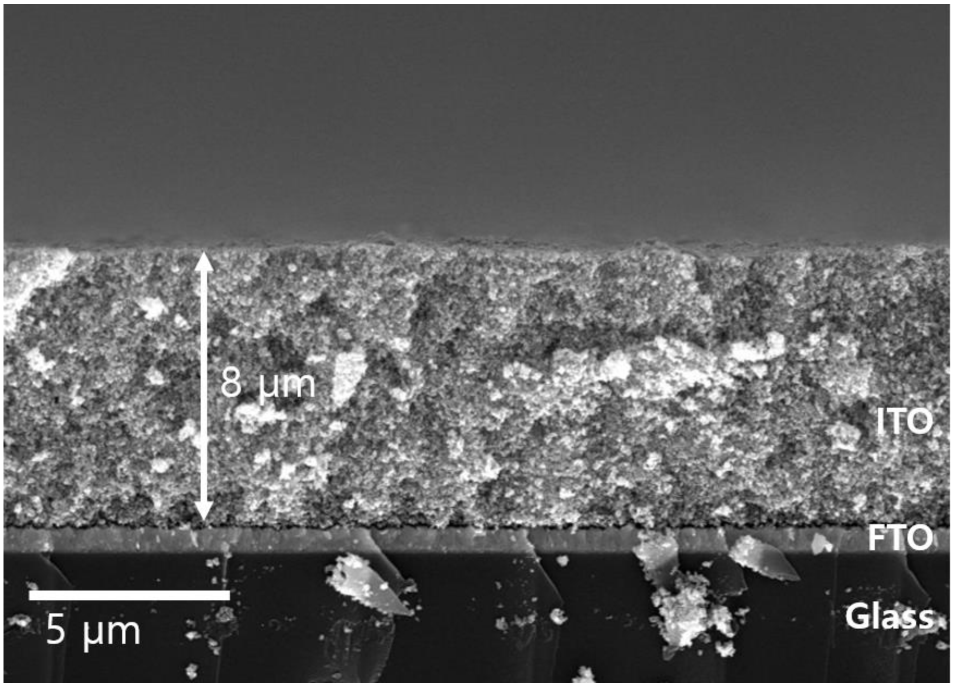

Figure S4 A cross-sectional image for a catalytic film, which was characterized by scanning electron microscopy (Inspect™ Scanning Electron Microscope F50, FEI)

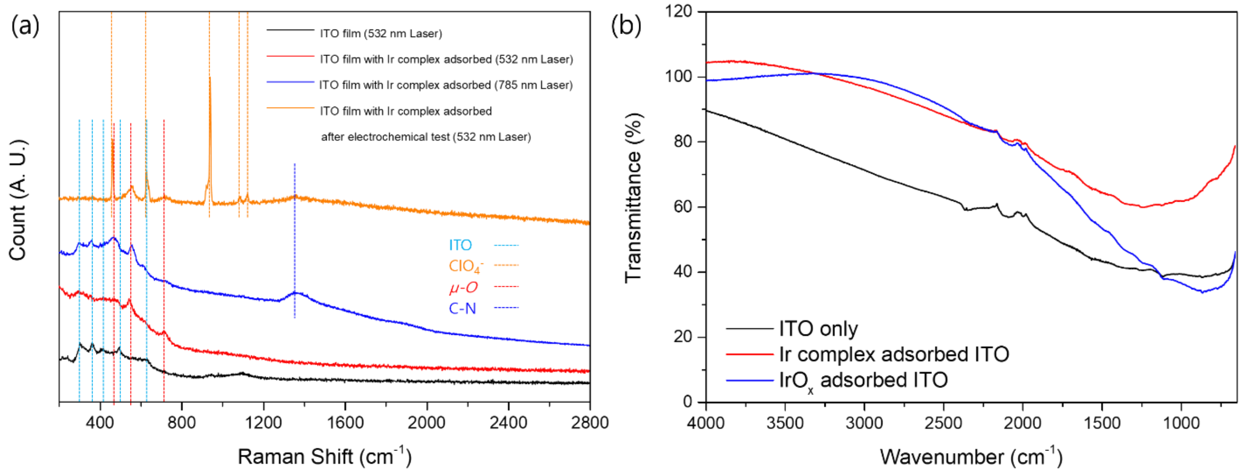

Figure S5. (a) E*x-situ* Raman spectroscopy results of ITO and Ir complex-adsorbed ITO films on FTO substrates (b) FT-IR spectroscopy results of Ir complex adsorbed ITO nanoparticles and IrO_x on ITO nanoparticles

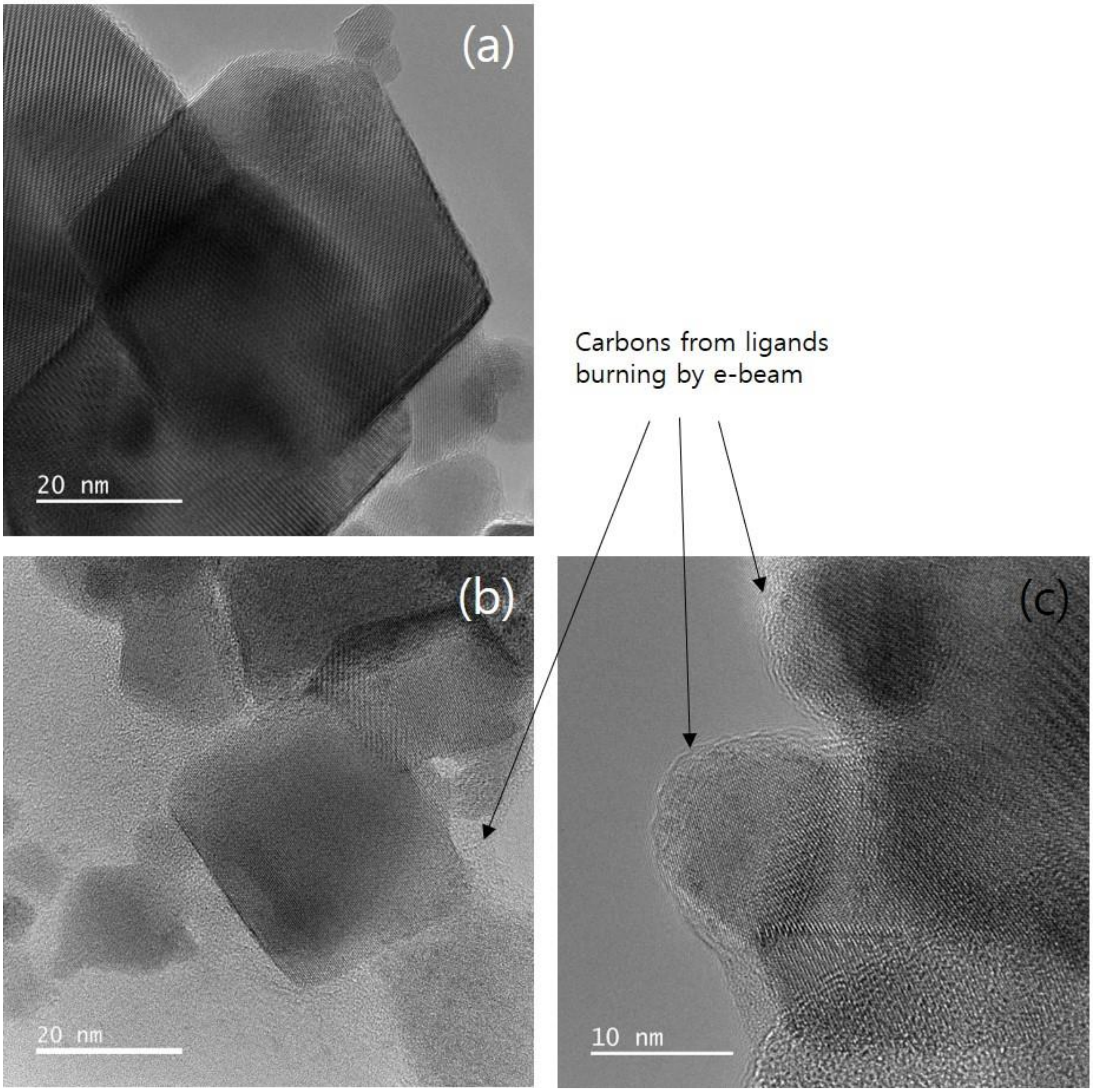

Figure S6. HR-TEM images of (a) bare ITO, (b) and (c) Ir complex adsorbed ITO nanoparticles. The complex was decomposing by the electron beam of TEM.

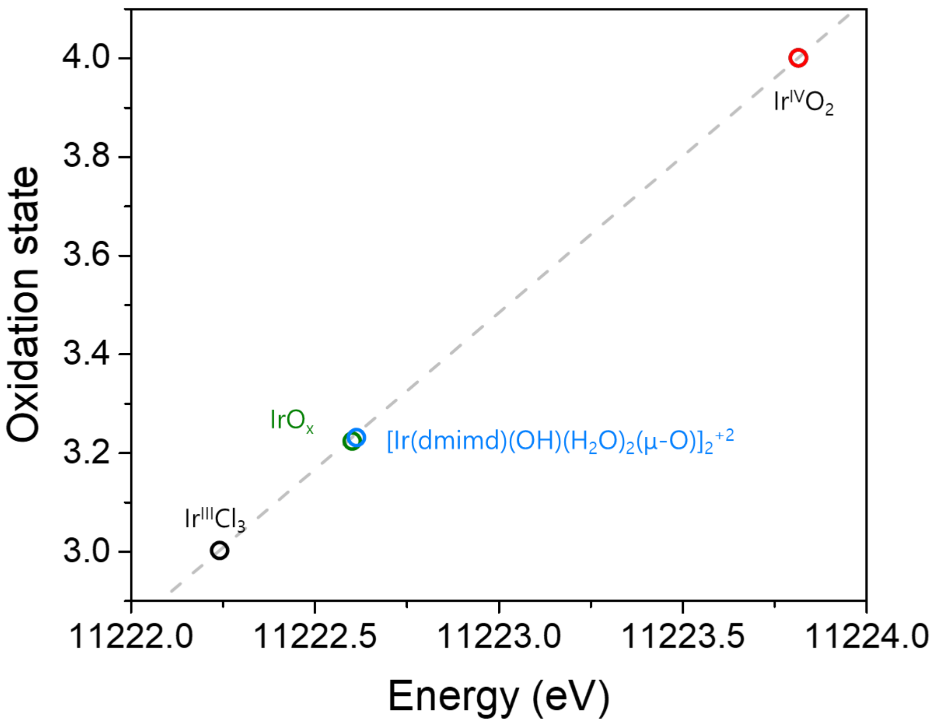

Figure S7. Calculated oxidation states of Ir complexes and Iridium oxides from XANES results

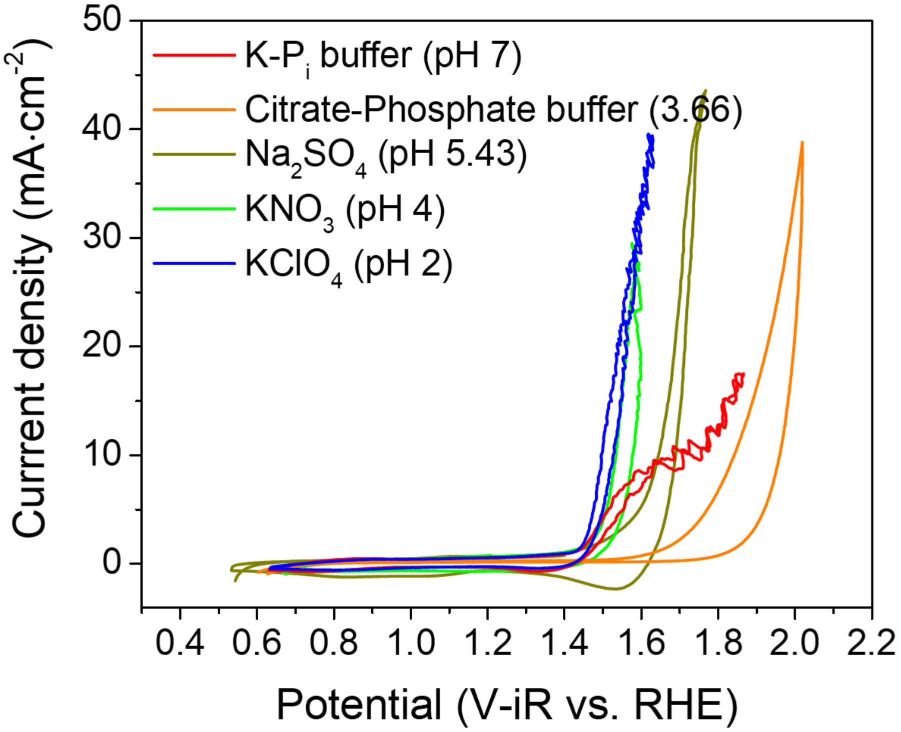

Figure S8. CV results of Ir complex/ITO on FTO substrates under different electrolyte conditions.

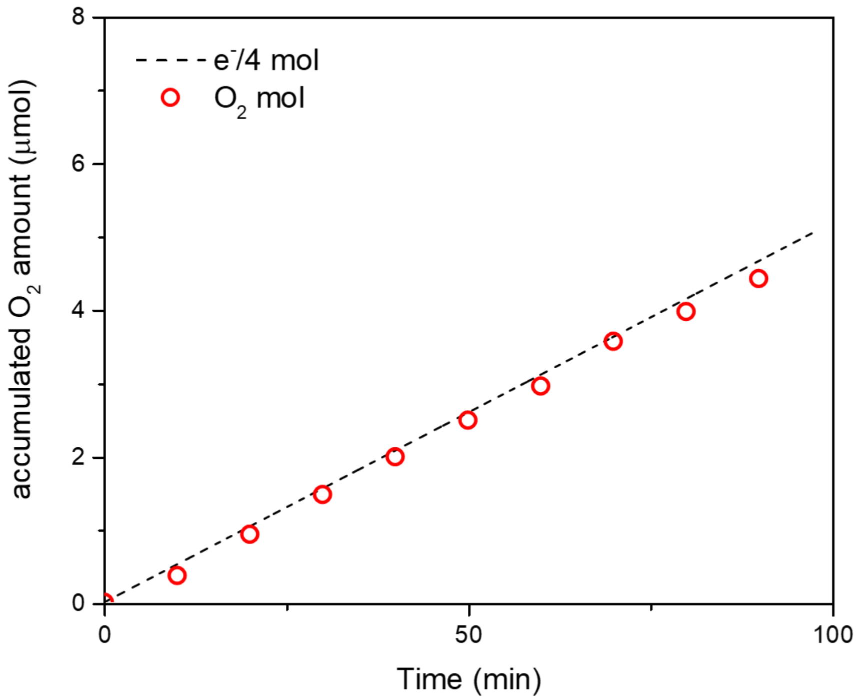

Figure S9. Evolved O_2 amounts of Ir complex adsorbed ITO/FTO electrodes for electrolysis.

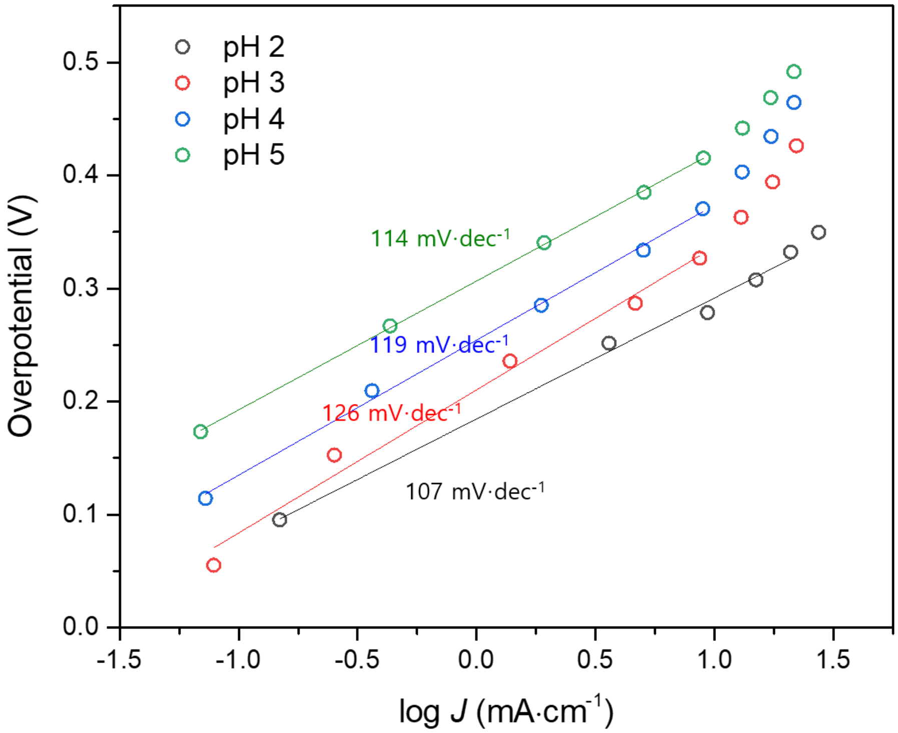

Figure S10. Tafel slope values of Ir complex-adsorbed ITO films on FTO substrates in 0.1 M $KClO_4$ with different pH conditions.

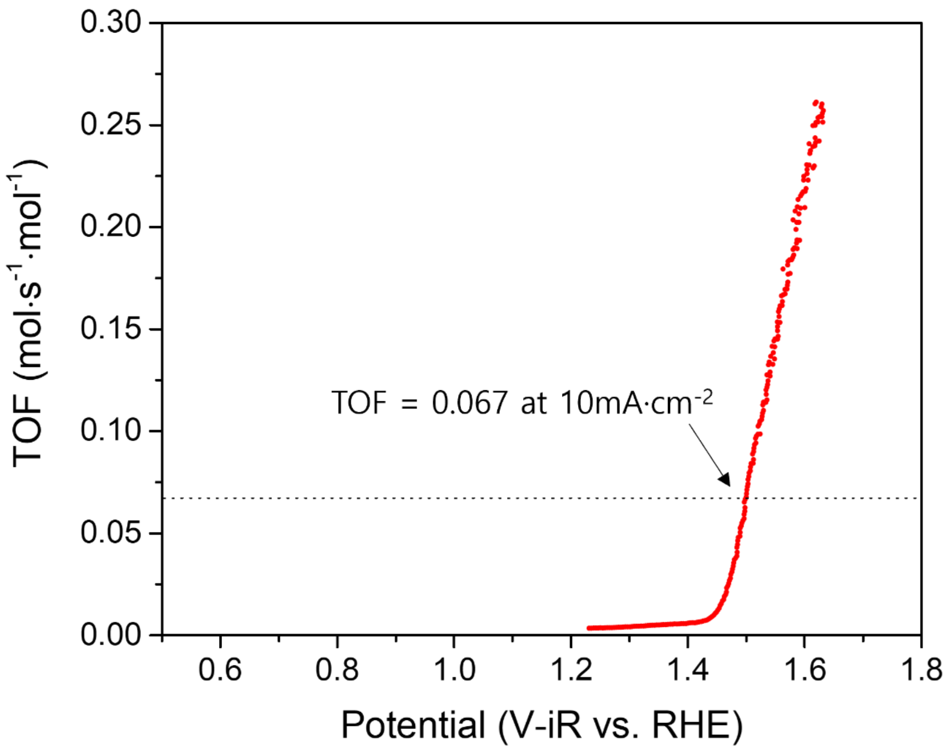

Figure S11. TOF on an Ir-complex-adsorbed electrode in a 0.1 M $KClO_4$ (pH 2) electrolyte.

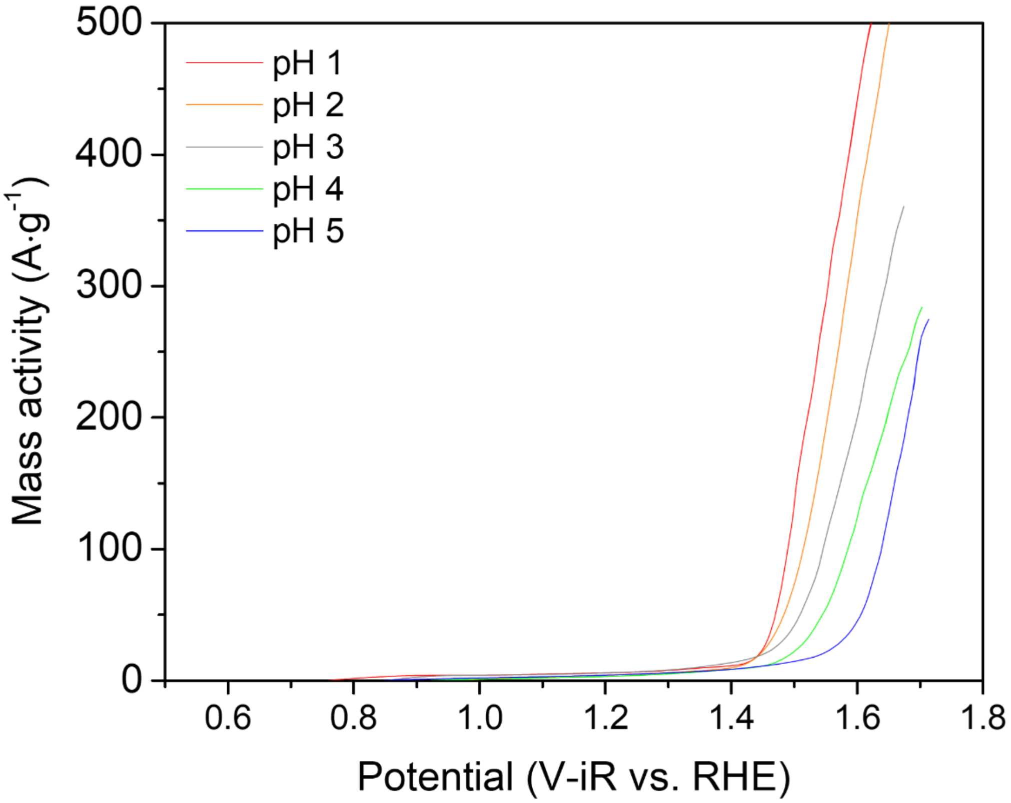

Figure 12. Mass activity of Ir complex/ITO in 0.1 M $KClO_4$ with different pH conditions

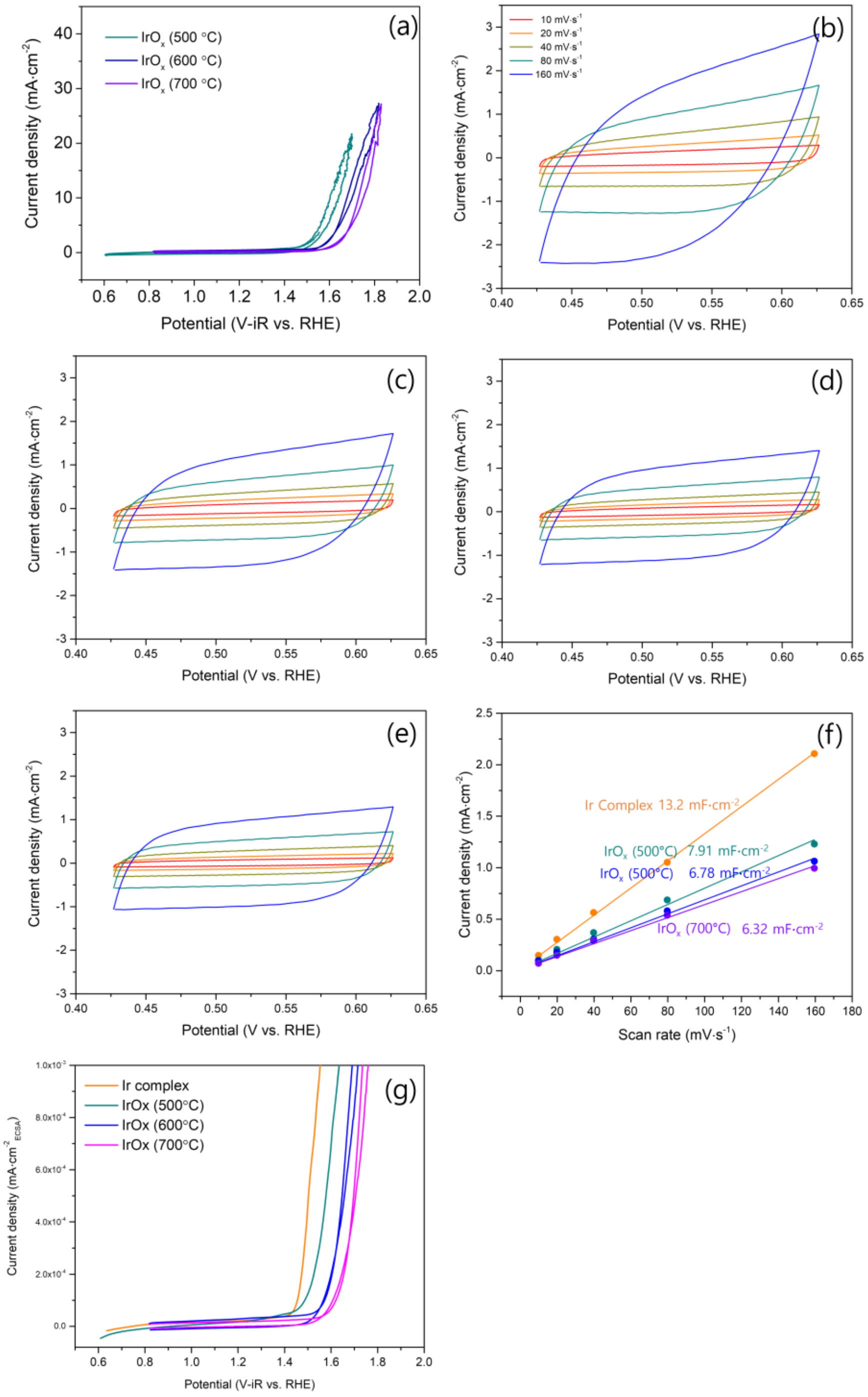

Figure S13. CV results of thermally prepared IrO_x/ITO on FTO substrates in 0.1 M $KClO_4$ (pH 2) under different annealing temperature conditions. The cyclic voltammetry results of (b) Ir complex, (c) IrO_x (500 °C annealed), (d) IrO_x (600 °C annealed), (e) IrO_x (700 °C annealed) in 0.1 M $KClO_4$ (pH 2). (f) Current density vs. Scan rate plot for Ir complex or IrO_x catalytic electrodes. (g) LSV or CV plots for current density vs. surface area. The surface area was estimated by C_{DL}/C_s, where C_{DL} is the double layer capacitance of catalytic electrodes, Cs is the specific capacitance of ITO (4.6 μF·cm^{-2})

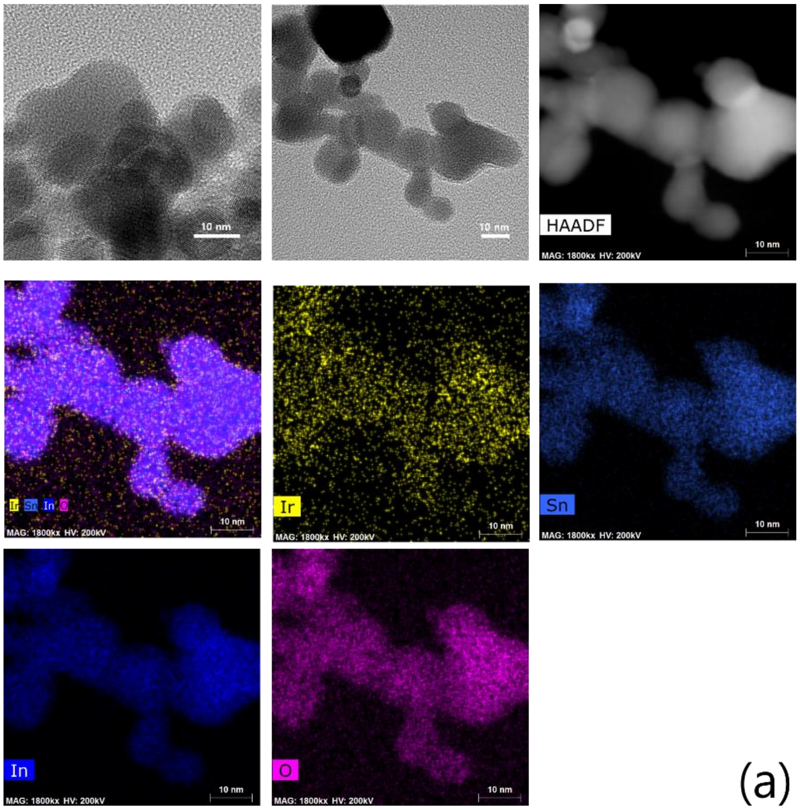
10 nm
10 nm
HAADF
10 nm
MAG: 1800kx HV: 200kV
Ir Sn In O
Ir
Sn
In
O

(a)

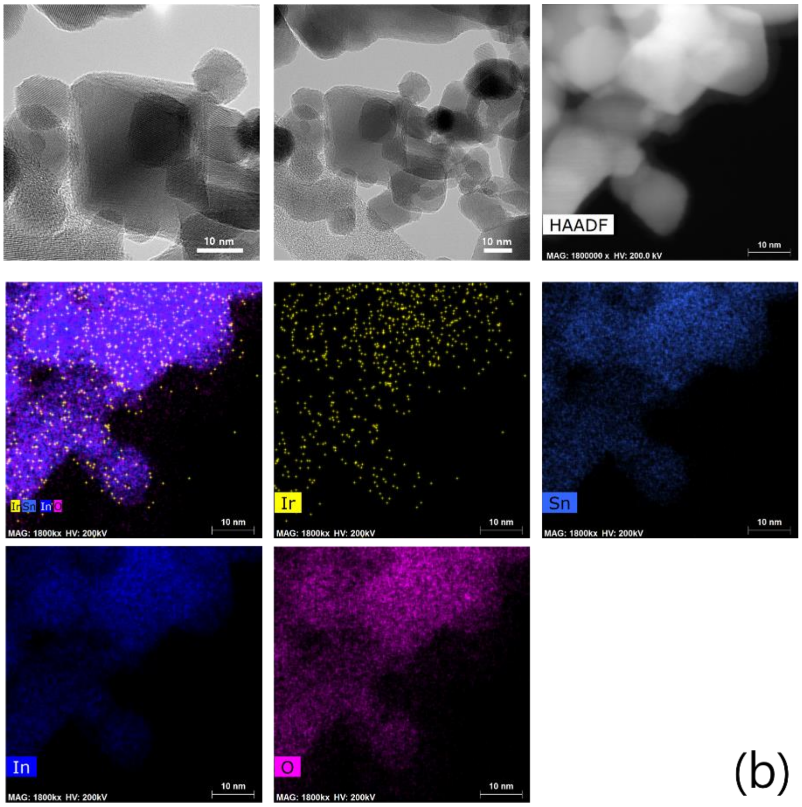
10 nm
10 nm
HAADF
10 nm
MAG: 1800000 x HV: 200.0 kV
Ir Sn In O
Ir
Sn
In
O
MAG: 1800kx HV: 200kV

(b)

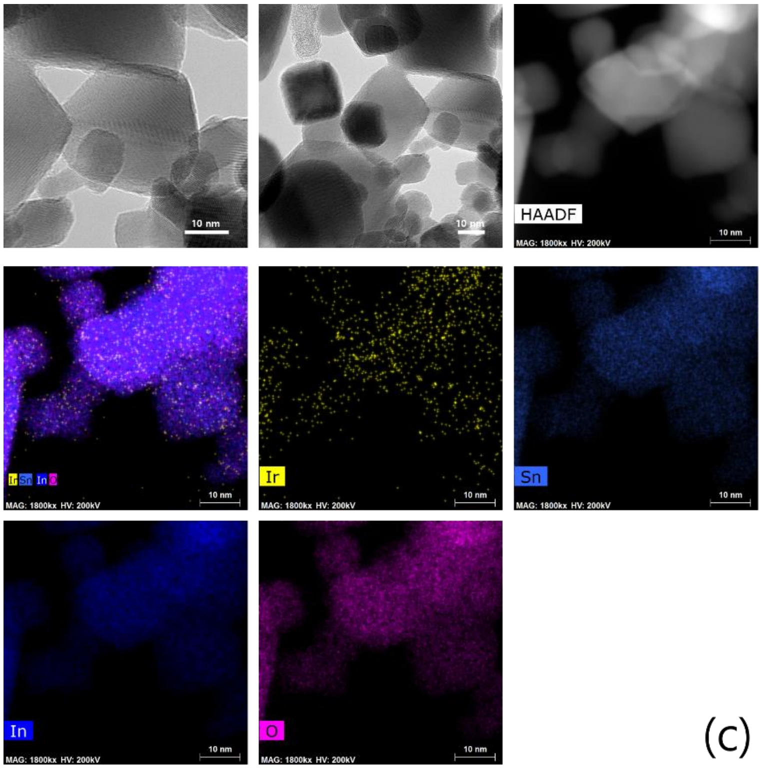

Figure S14. HR-TEM and EDS mapping images of thermally prepared IrO_x on ITO nanoparticles at (a) 500°C, (b) 600°C, and (c) 700°C

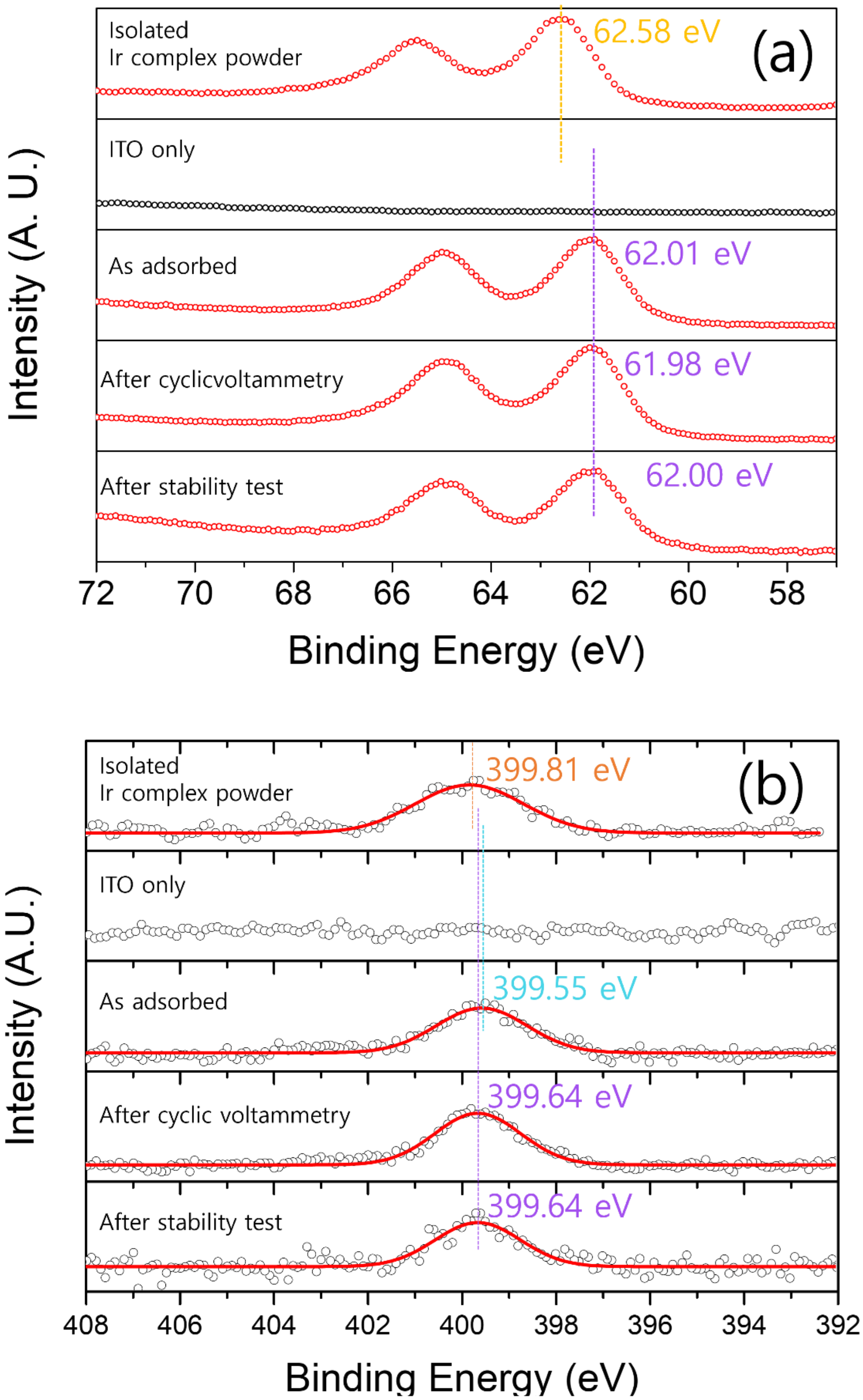

Figure S15. XPS spectra of (a) Ir 4*f* and (b) N 1*s* on Ir complexes (as-adsorbed), after cyclic voltammetry or stability tests (in 0.1M $KClO_4$ (pH 2)).

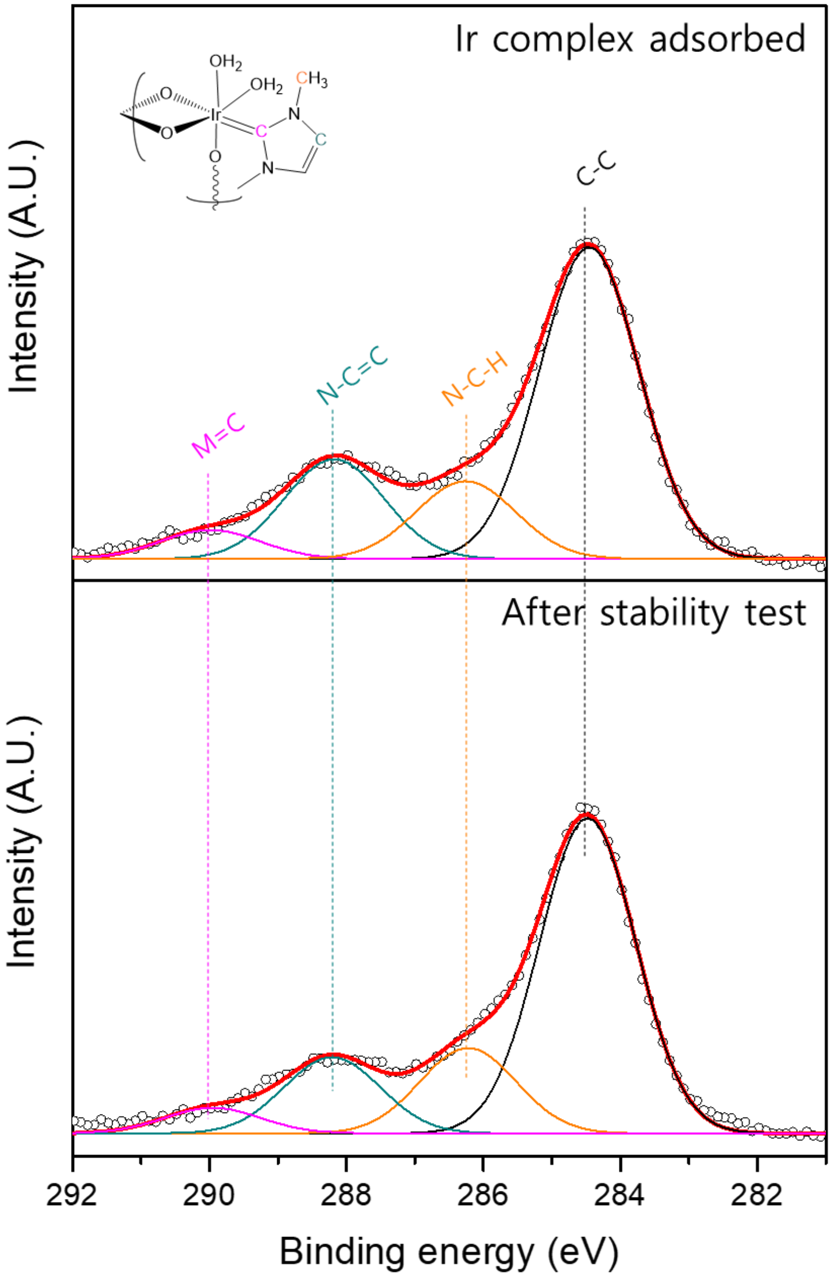

Figure S16. XPS spectra of C 1s from an Ir complex adsorbed ITO film before stability tests (top) and after stability tests (bottom) in 0.1 M $KClO_4$ (pH 2). Peaks were assigned by reference.

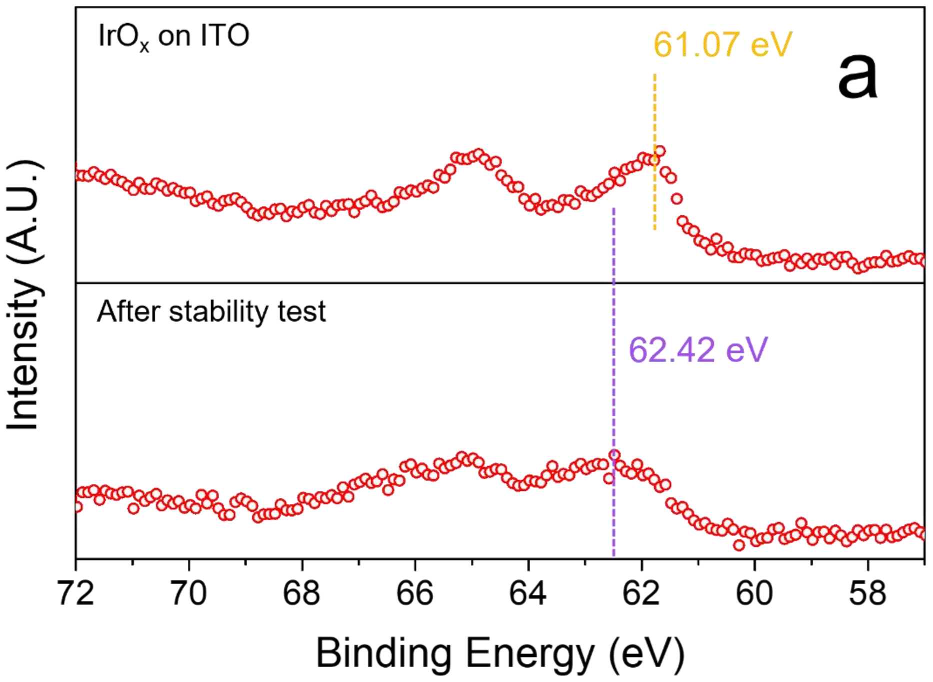

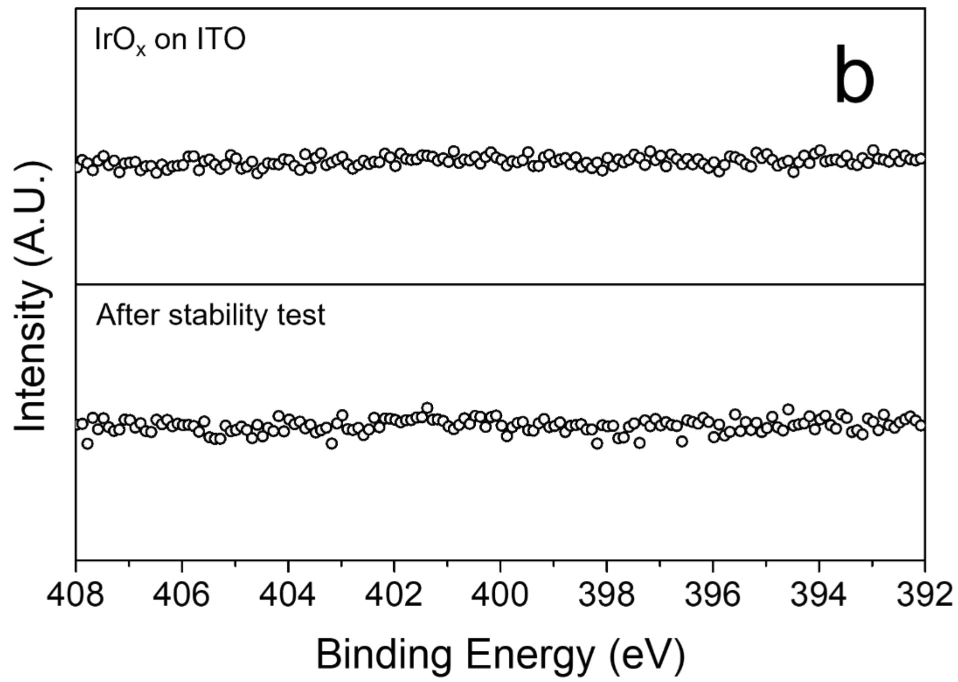

Figure S17. XPS spectra of (a) Ir 4*f* and (b) N 1*s* on IrO_x/ITO films before/after stability tests in 0.1 M $KClO_4$ (pH 2).

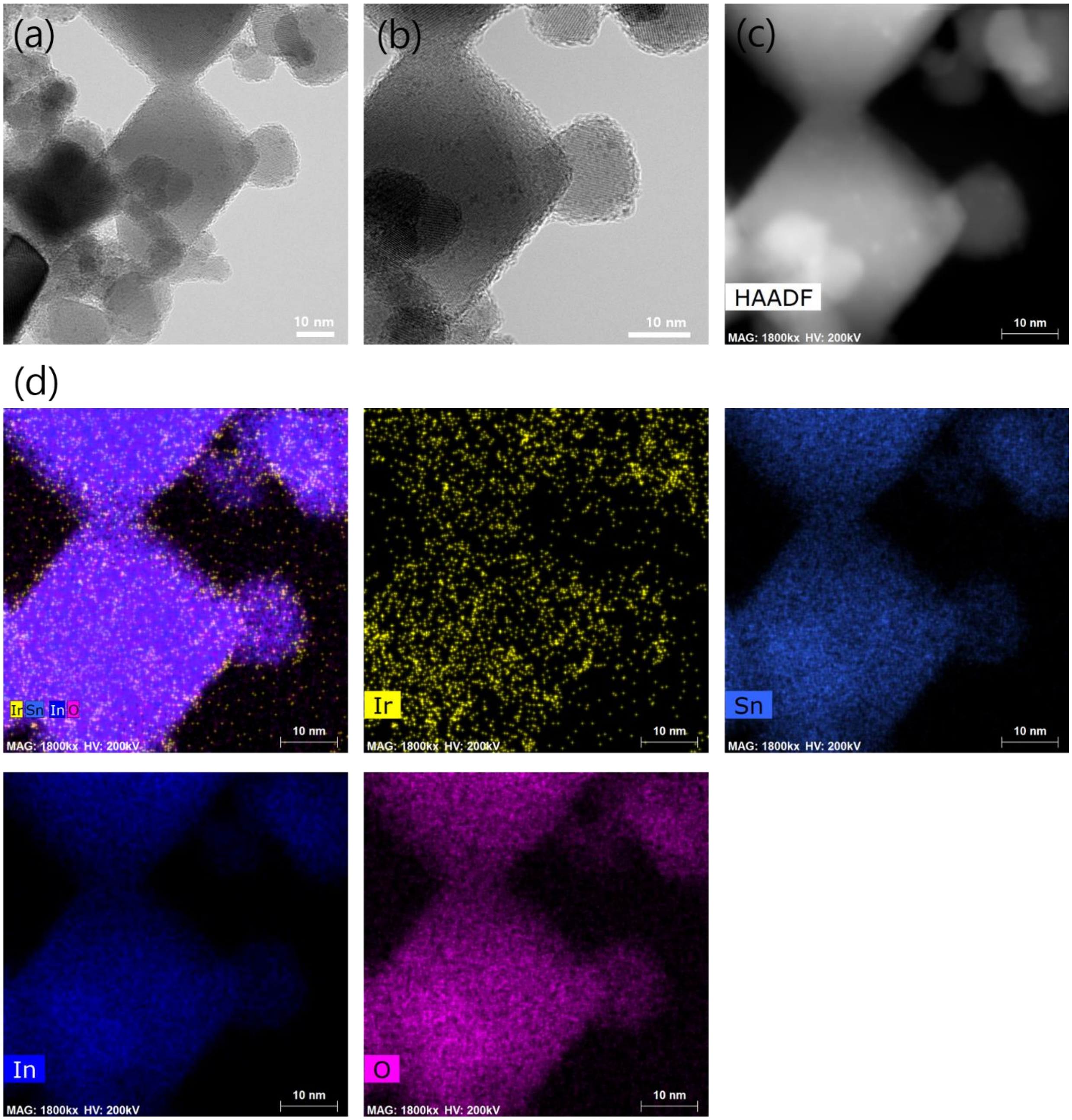

Figure S18. (a), (b), and (c) HR-TEM images and (d) EDS mapping images of Ir complexes-absorbed ITO after stability tests in 0.1M KClO4 (pH 2).

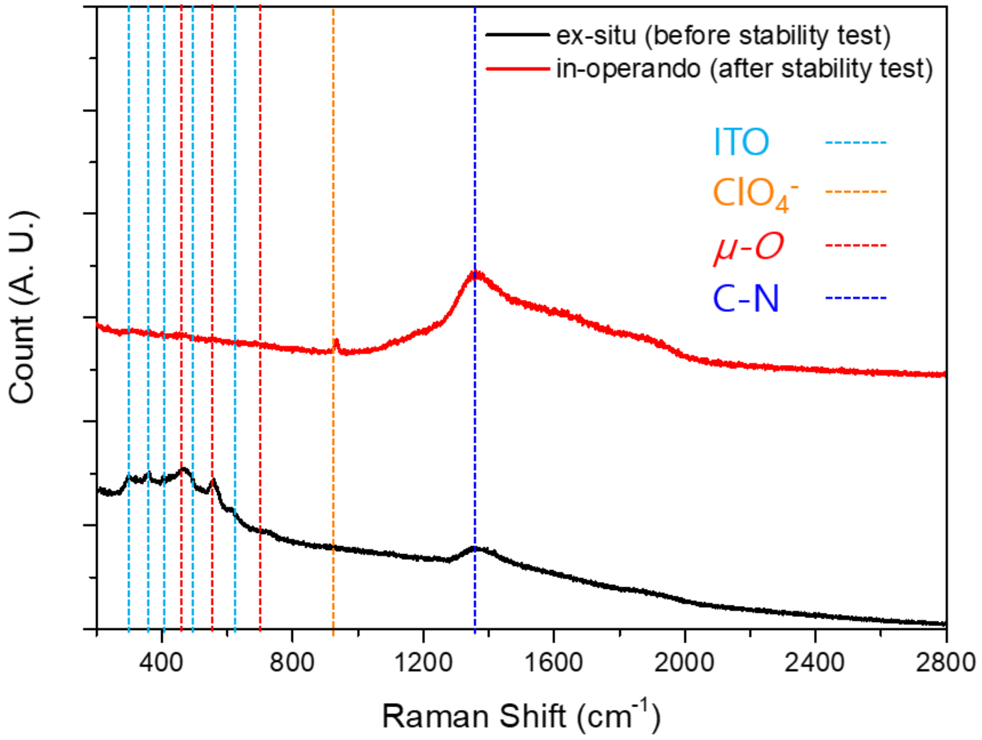

Figure S19. Comparison of *ex-situ* Raman spectrum of Ir-complex-adsorbed ITO particle/FTO films and its *in-operando* Raman spectrum with a 785 nm laser after stability tests in 0.1 M $KClO_4$ (pH 2)

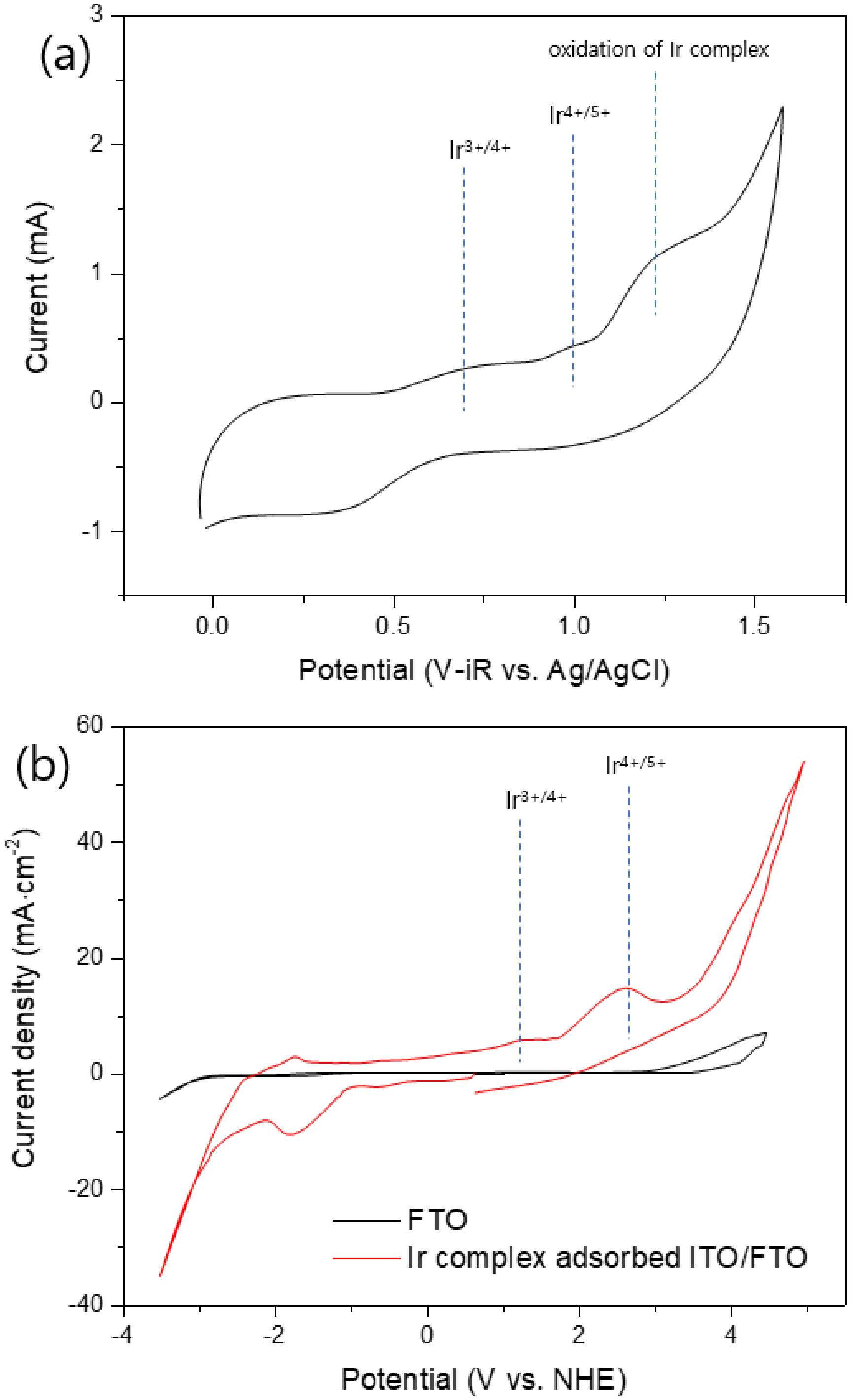

Figure S20. (a) Cyclic voltammetry of a bare ITO film on FTO substrates in a 1 mM Ir complex dissolved solution with a 50 mV·s^{-1} scan rate. (b) Cyclic voltammetry of an Ir complex adsorbed ITO film on FTO substrates in a 0.1 M tetrabutylammonium perchlorate acetonitrile solution with a 100 mV·s^{-1} scan rate. The potential was calibrated by ferrocene.

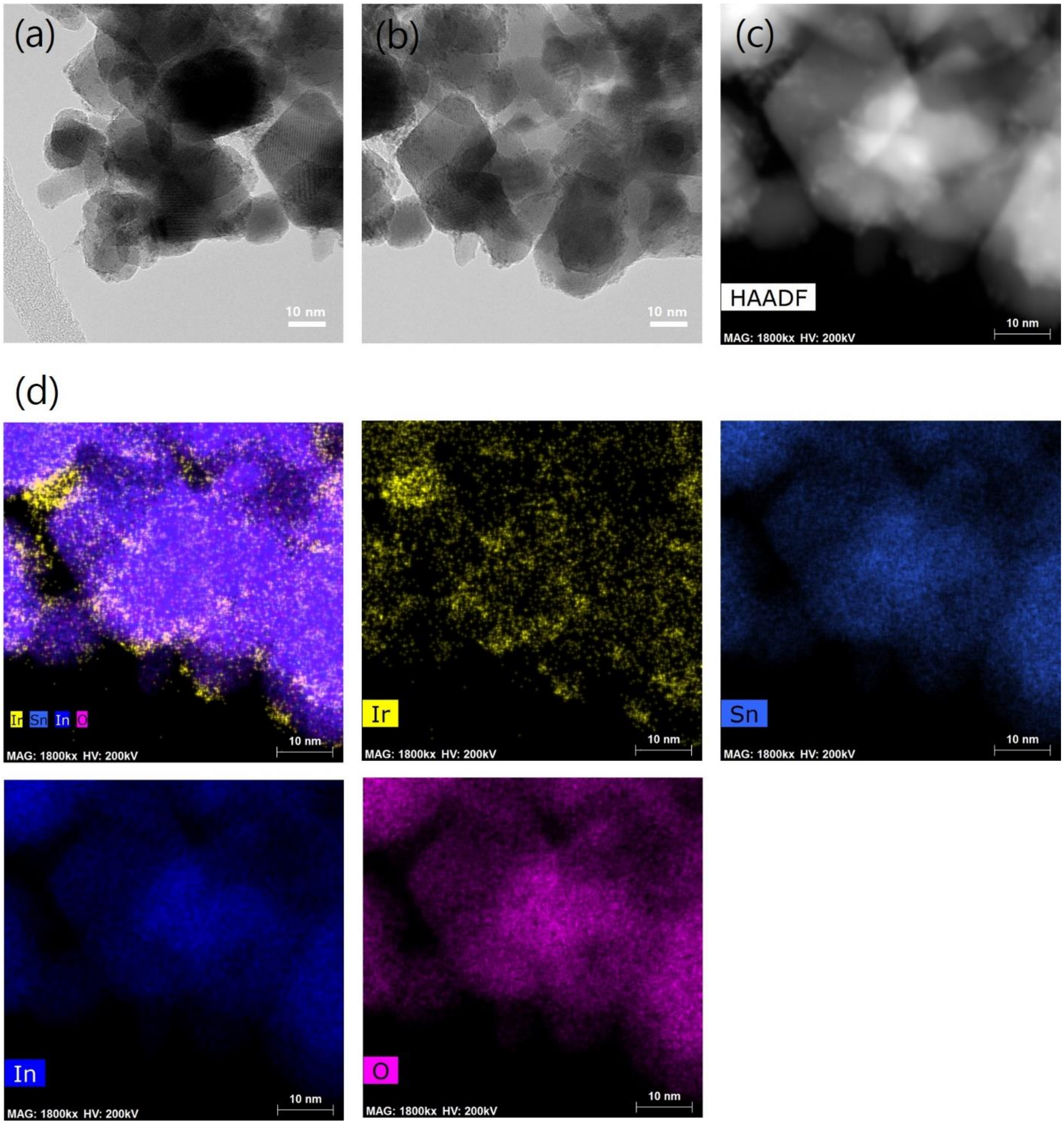

Figure S21. (a), (b), and (c) HR-TEM images and (d) EDS mapping images of bare ITO after applying potential in the 1 mM Ir complex dissolved electrolyte.

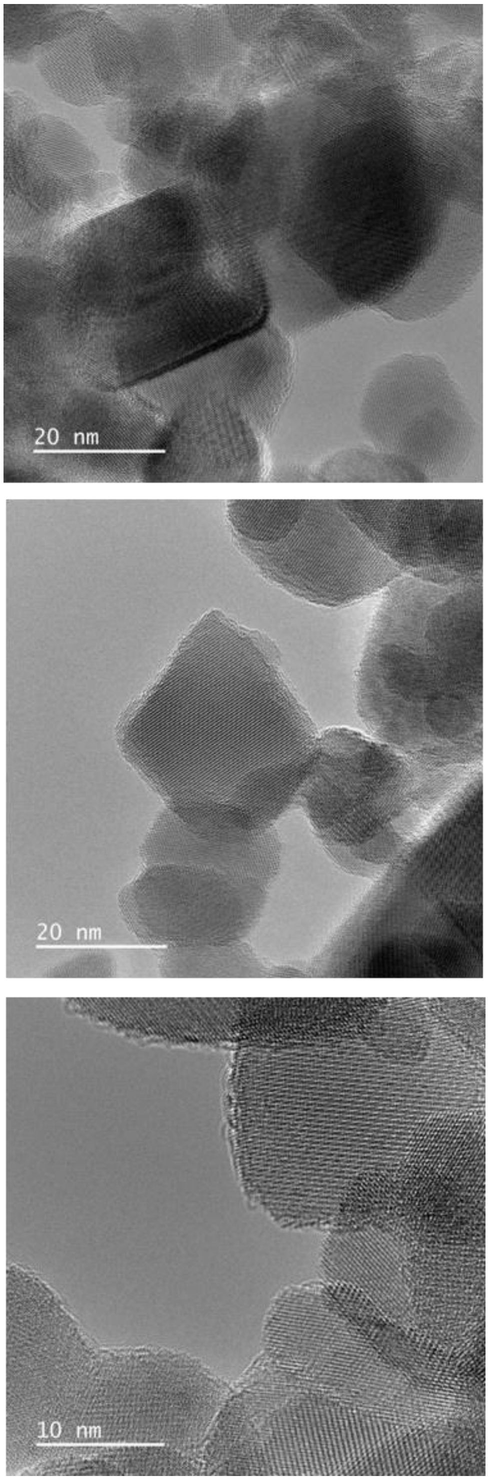

Figure S22. HR-TEM images of IrO_x on ITO nanoparticles after stability tests in 0.1 M $KClO_4$ (pH 2).

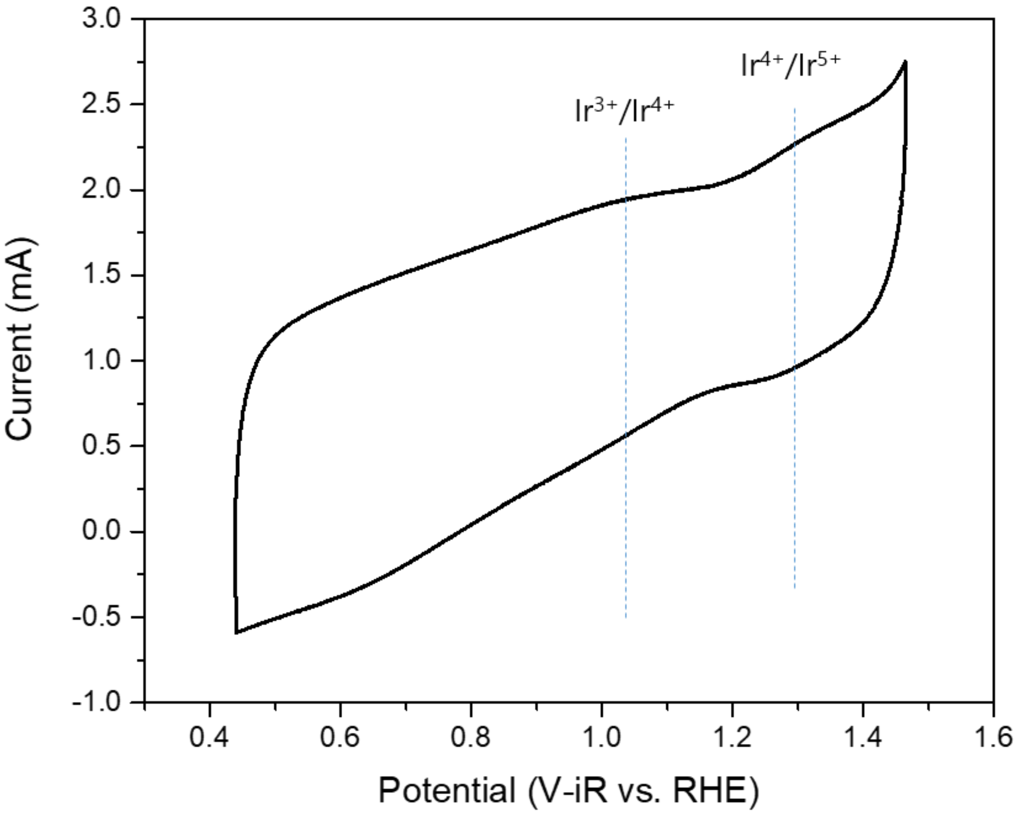

Figure S23. A cyclic voltammetry result of a IrO_x/ITO film with a 50 mV·sec^{-1} scan rate in 0.1 M $KClO_4$ (pH 2)

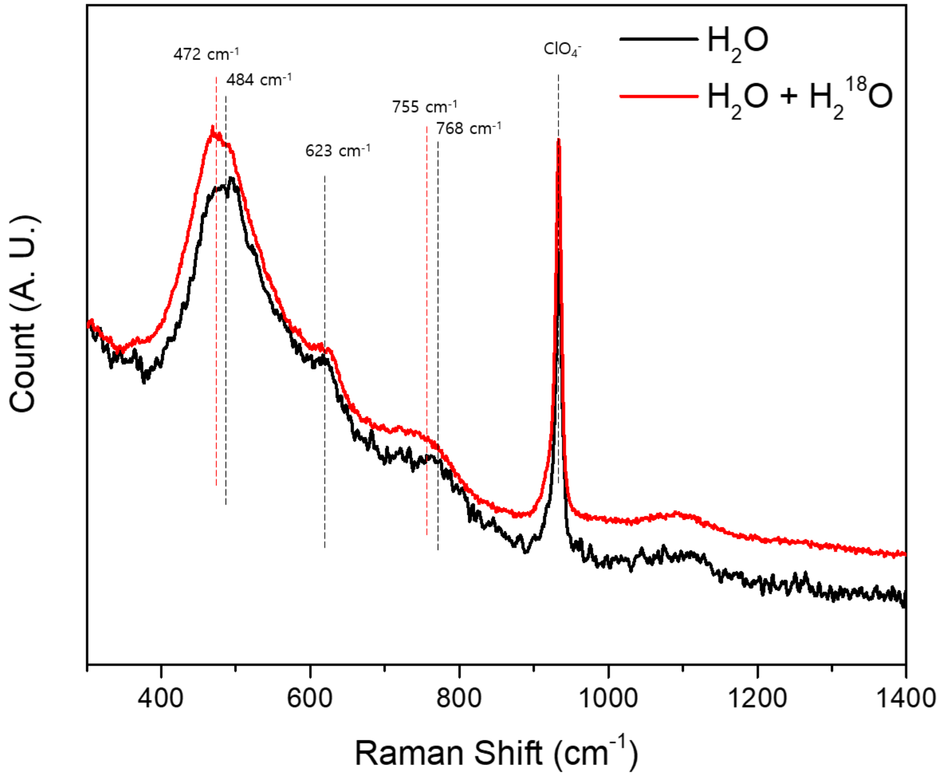

Figure S24. In-operando Raman spectra with different electrolytes, 0.1 M $KClO_4$-H_2O (black line) and 0.1 M $KClO_4$-$H_2{}^{18}O$+$H_2{}^{16}O$ (5 : 3 volume ratio) (red line), respectively. The applied potential was 1.53 V vs. RHE

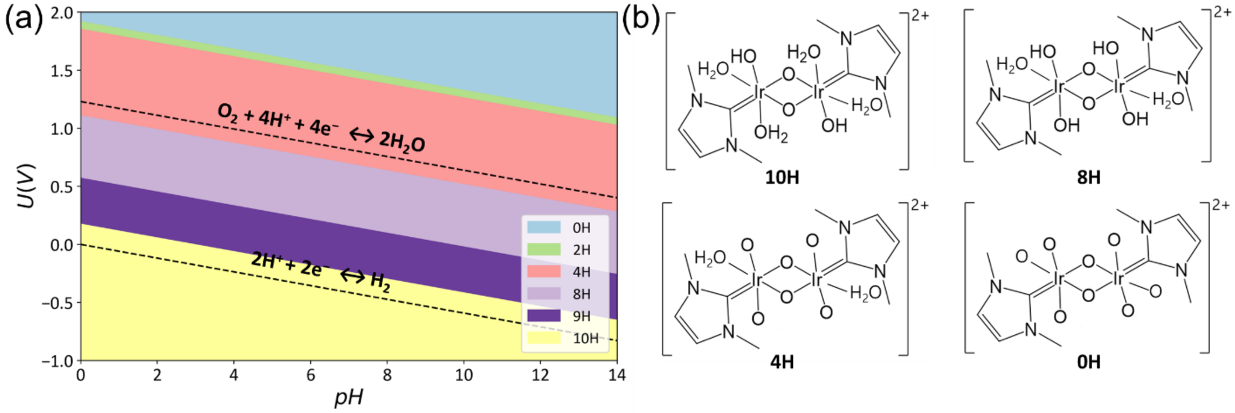

Figure S25. (a) The number of protons remaining on the Ir-imidazole molecule under different conditions (applied potential and pH). The two dashed lines indicate the equilibrium lines of OER and HER. (b) Schematic drawings of various Ir-imidazole molecules presented in (a).

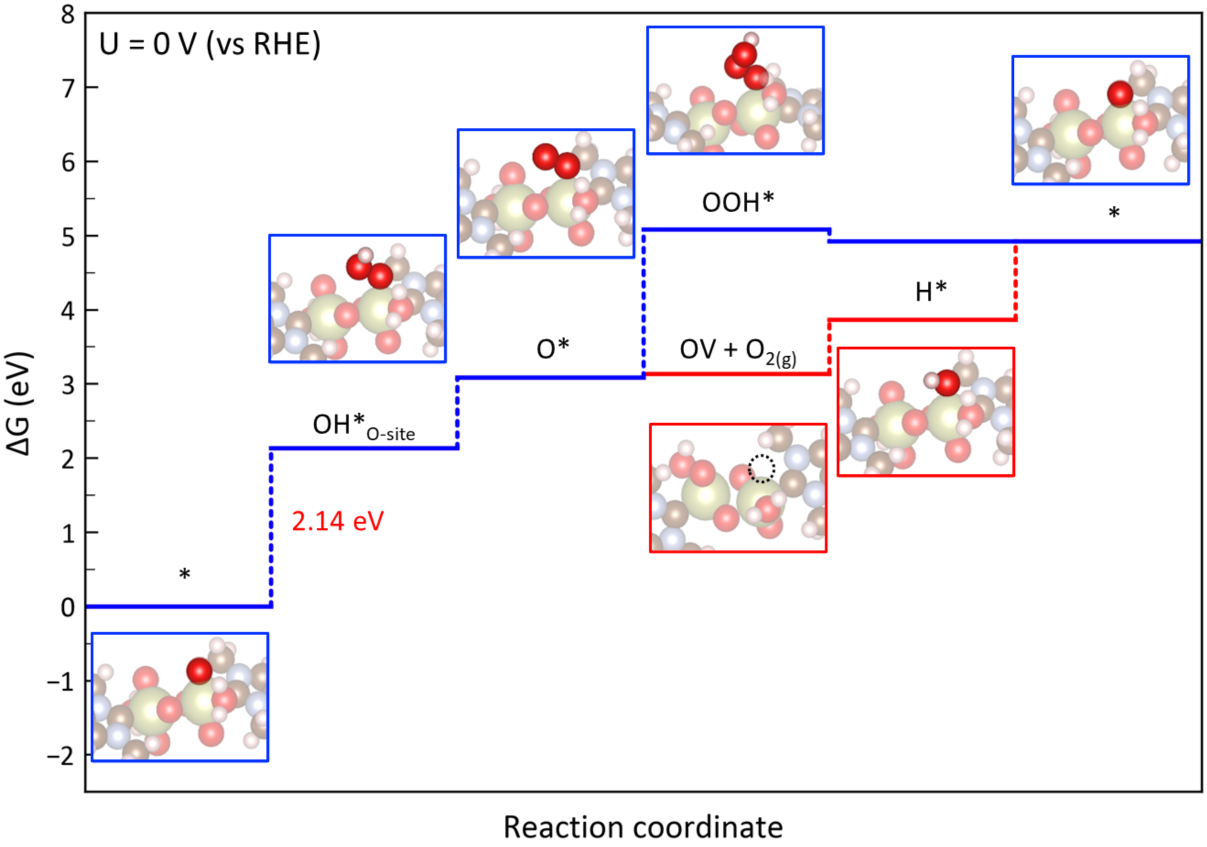

Figure S26. Free energy diagram for OER on the partially deprotonated Ir-imidazole-4H molecule (only four protons remaining) at U = 0 V vs RHE. The value of the reaction free energy for a rate-determining step is shown in red.

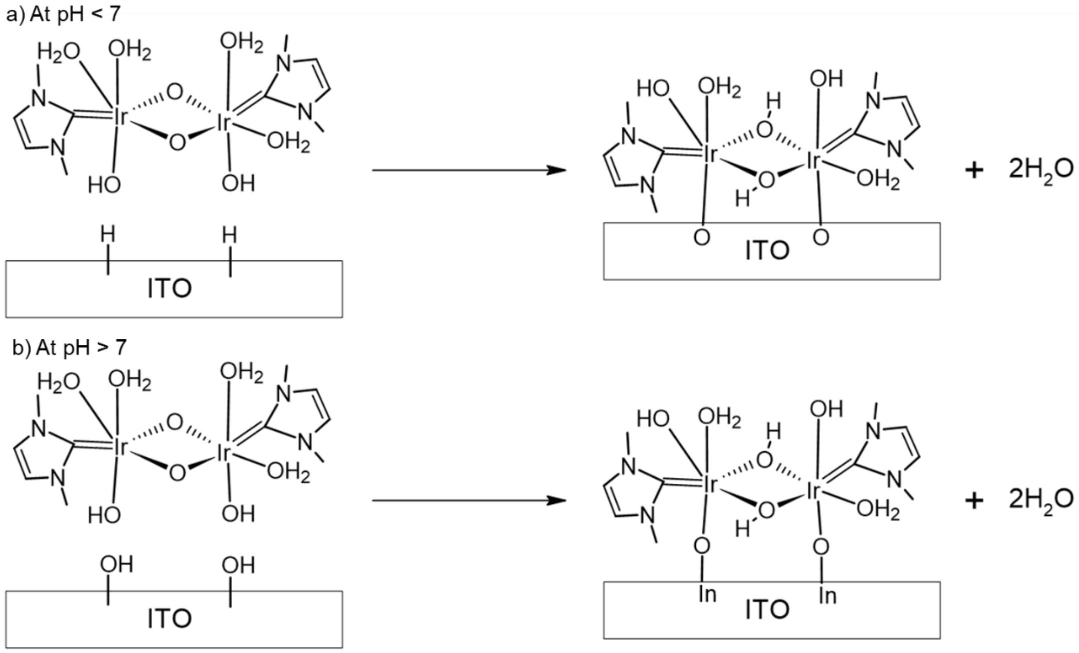

Figure S27. Different anchoring reactions of fully protonated Ir-imidazole molecules on ITO at different surface coverages that depend on pH.

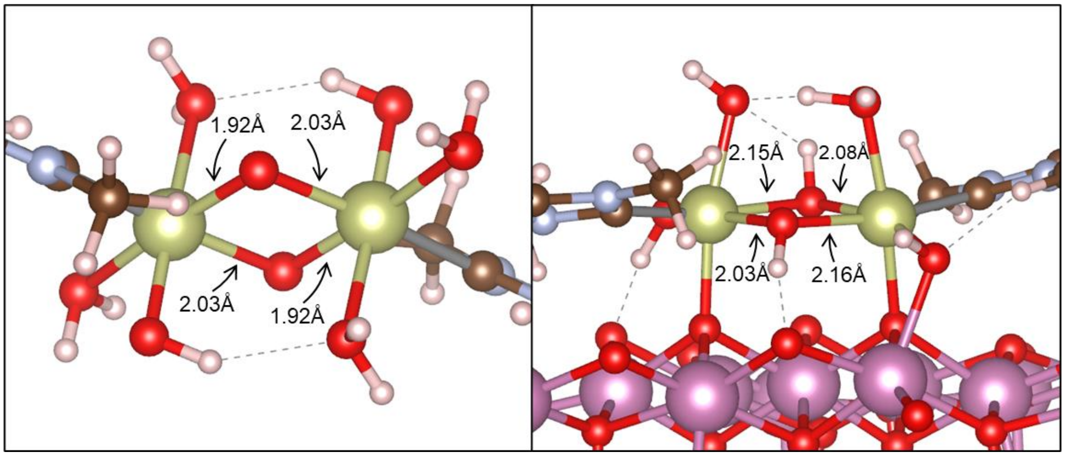

Figure S28. Changes in the bond lengths of a fully protonated Ir complex before and after anchoring on ITO surface.

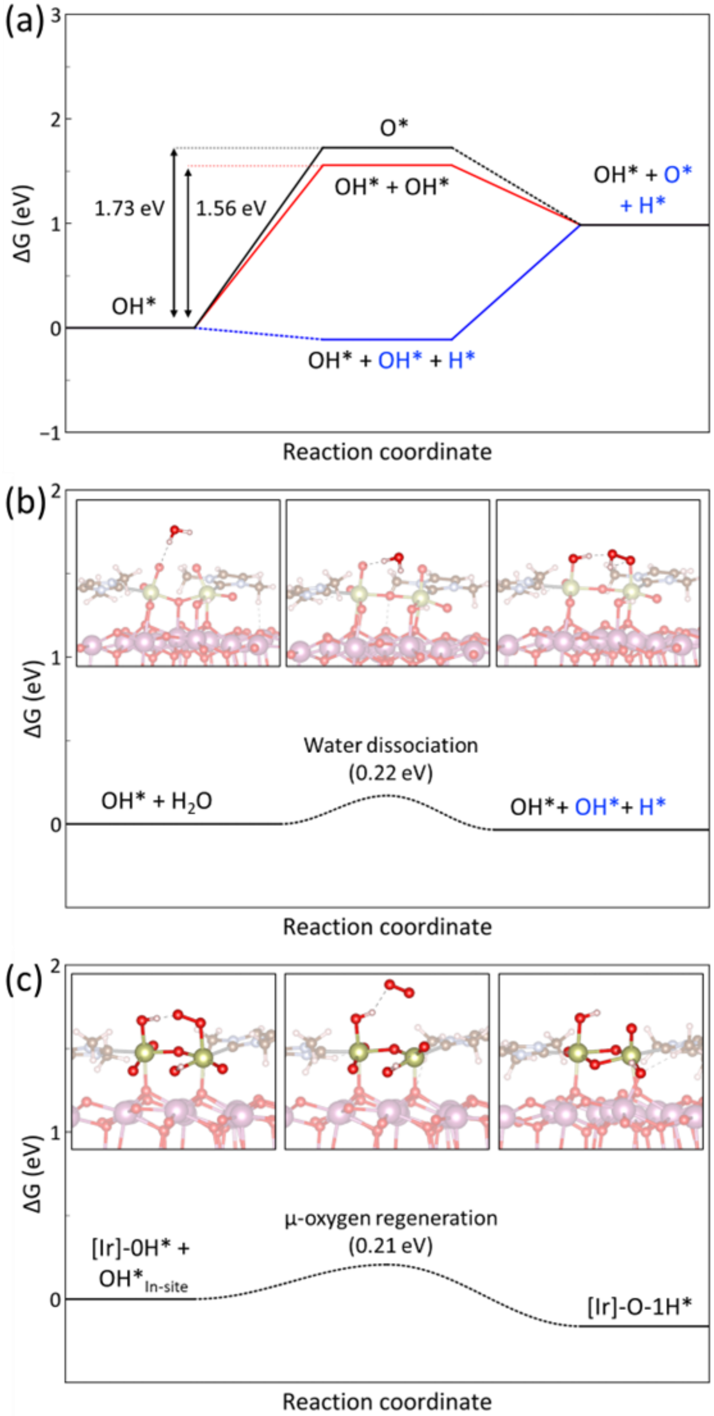

Figure S29. (a) A Gibbs free energy diagram for three different routes after OH* is formed on the fully deprotonated Ir complex anchored on ITO (111) surface: (1) Electrochemical oxidation of the formed OH* to O* (OH* → O* + H^+ + e^-) is shown in black; (2) Another OH* formation via electrochemical water dissociation (H_2O → OH* + H^+ + e^-) is shown in red; and (3) Thermochemical water dissociation (H_2O → $OH*_{O\text{-site}}$ + $H*_{O\text{-site}}$) is shown in blue. Detailed reaction energetics for thermochemical water dissociation and regeneration of the μ-O bonds via O_2 desorption are shown in (b) and (c), respectively.

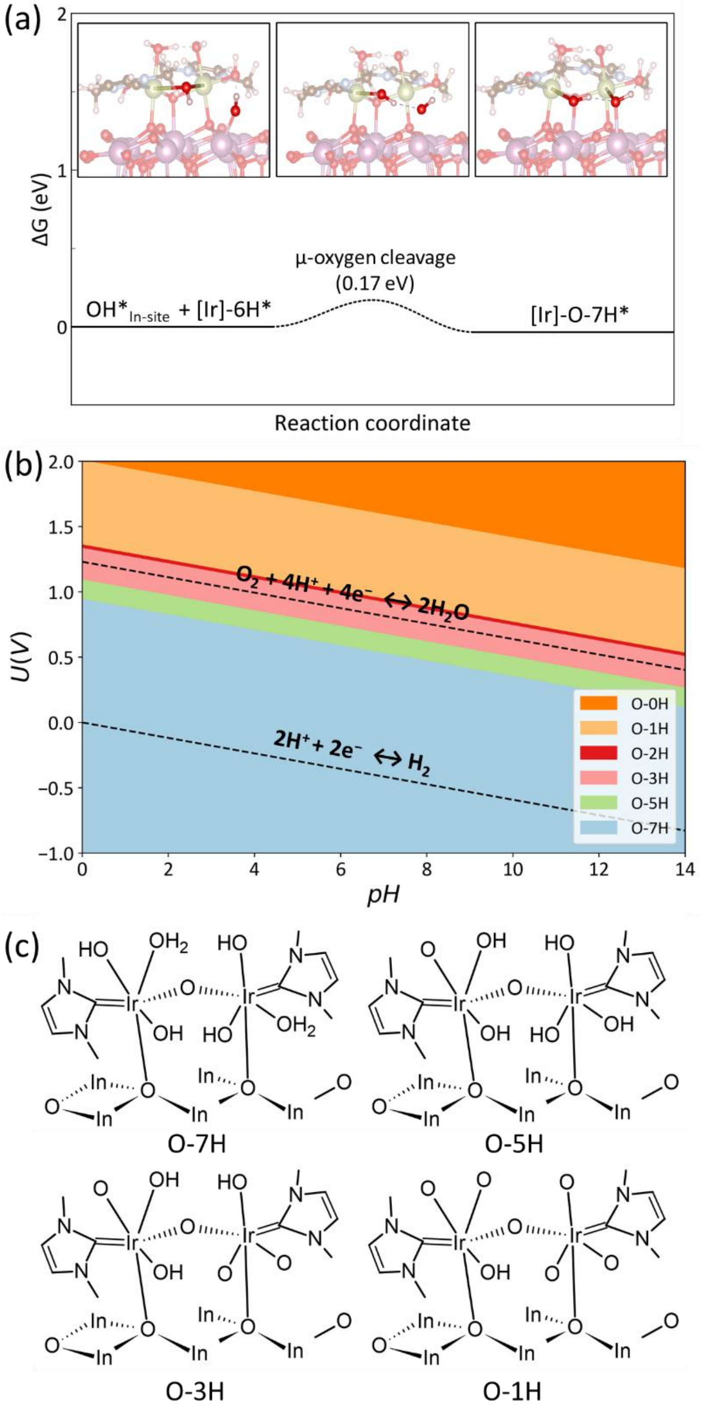

Figure S30. (a) A detailed reaction pathway for the thermochemical reaction of OH* and Ir complex–6H each other to result in Ir complex–O–7H (b) The number of protons remaining on the OH* added Ir complex anchored on ITO surface under different conditions (applied potential and pH). (c) Schematic drawings of the OH* added Ir complexes anchored on ITO surface presented in (b).

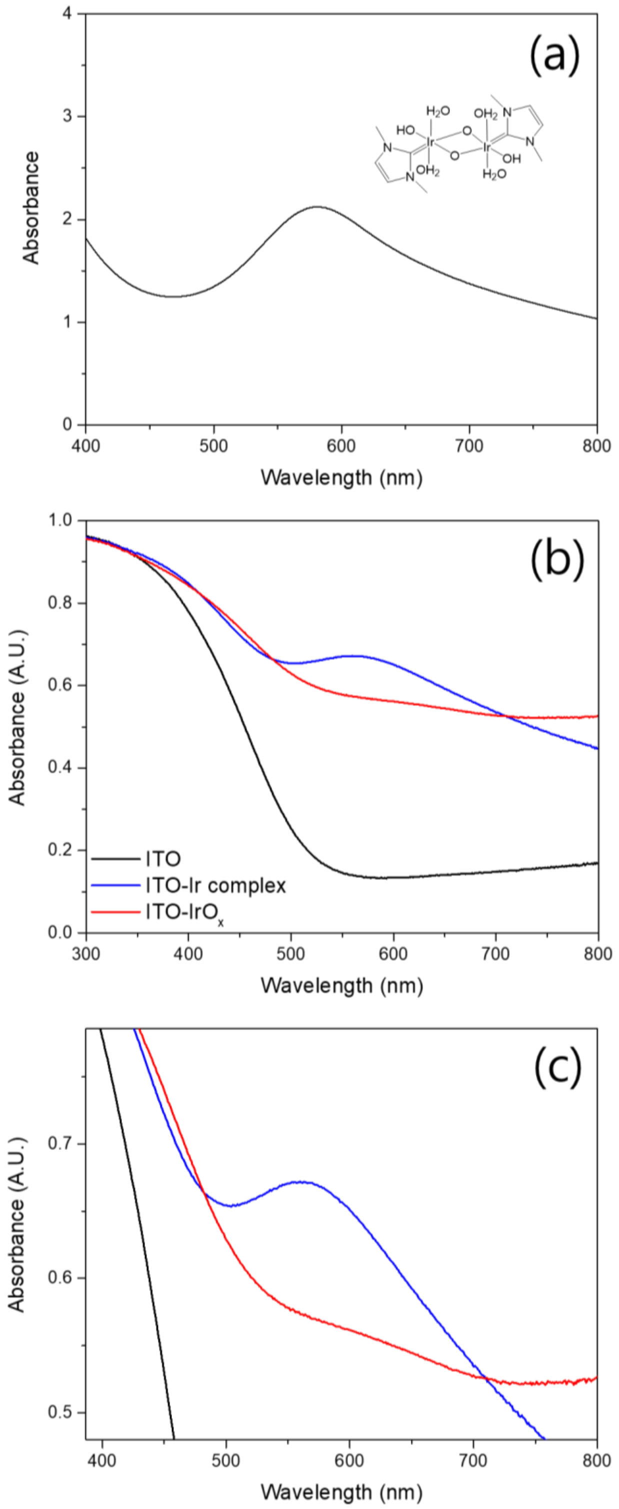

Figure S31. (a) Absorbance spectrum of 1 mM of an aqueous Ir complex solution. (b) Absorbance spectra of ITO, Ir complex-adsorbed ITO, IrO_x-ITO films on FTO substrates. (c) Is scale-zoomed graphs of (b) for easily comparing two absorption peaks. The results were obtained by an UV-Vis-NIR spectrometer with an integrated sphere (Cary 5000, Agilent)

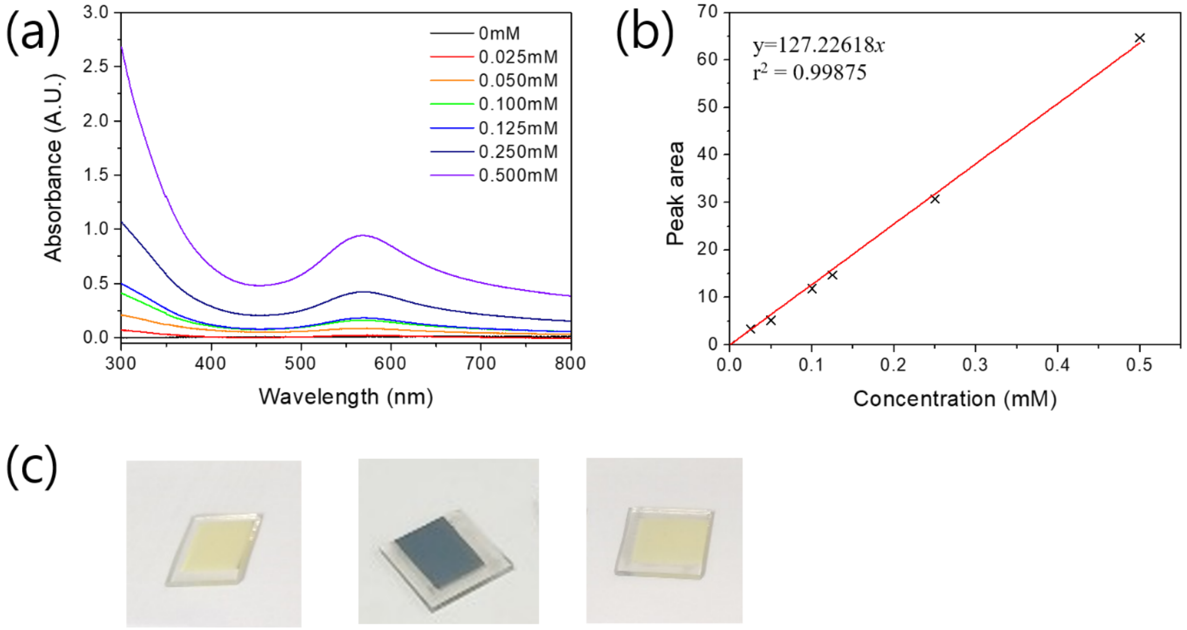

Figure S32. (a) Absorbance of $[Ir(dmimd)(OH)(H_2O)_2(\mu\text{-}O)]_2^{2+}$ at different concentrations. (b) Calibration curve of peak area vs. concentration of $[Ir(dmimd)(OH)(H_2O)_2(\mu\text{-}O)]_2^{2+}$ solutions. (c) Photograph images of an ITO film on FTO (left), after chemisorption of Ir complexes (center), and after desorption of the Ir complexes from the ITO film (right)

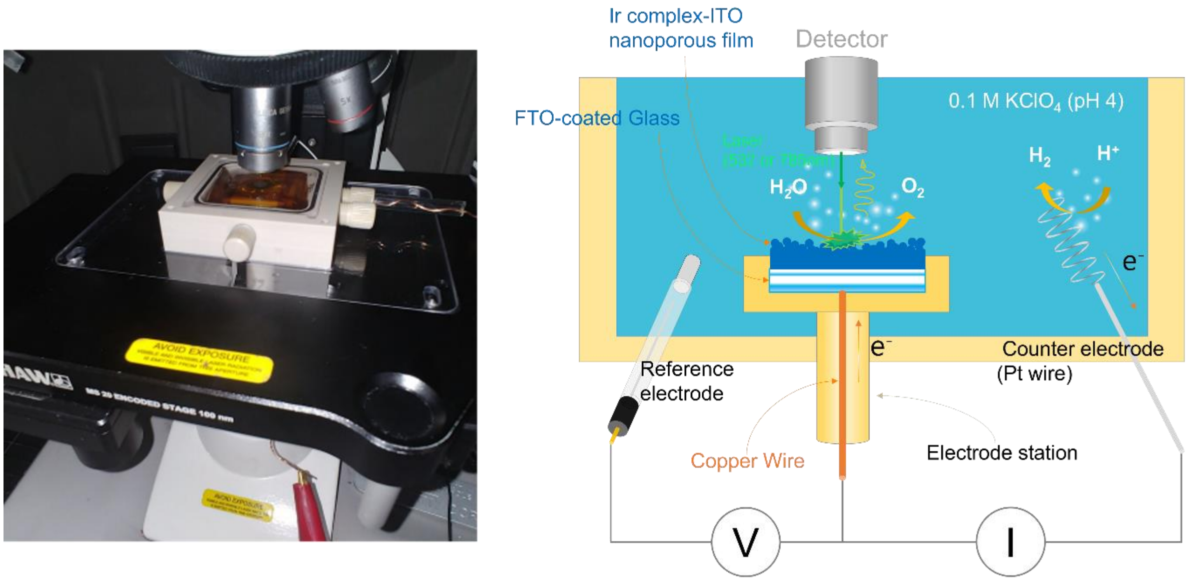

Figure S33. A photograph of a set-up and a schematic image of an electrochemical cell in the Raman microscope for *in-operando* measurement.

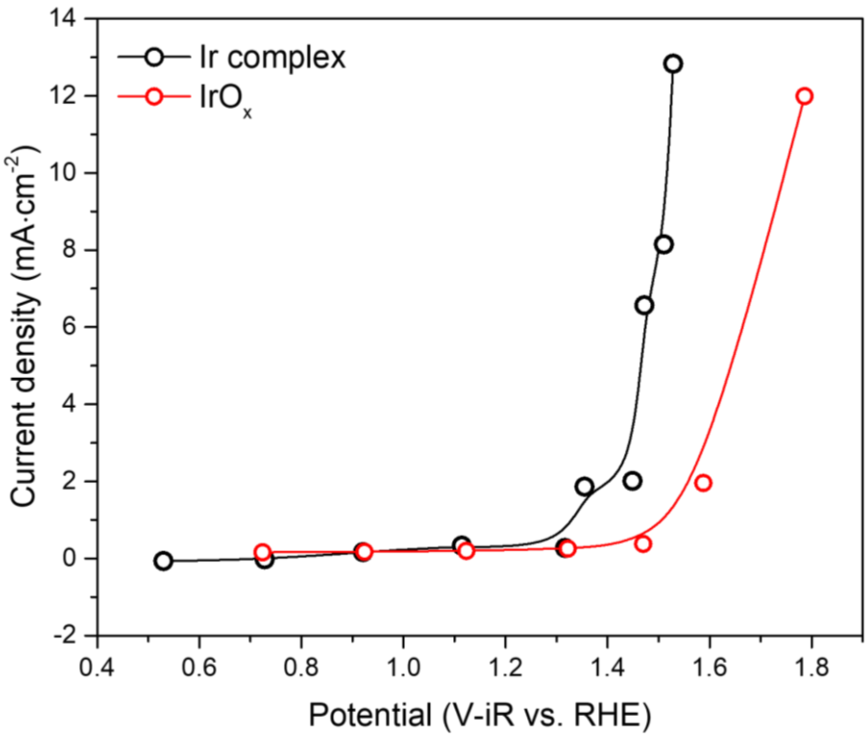

Figure S34. Potential vs. steady-state current results during *in-situ* Raman spectroscopy in 0.1 M $KClO_4$ (pH 2) electrolyte conditions. Unfilled circles indicate steady-state current density during Raman spectroscopy measurement.

Table S1. A comparison table for immobilized molecular complexes and IrO_x-related electrocatalysts for the electrochemical OER.

Catalyst	Conducting Support	Overpotential (mV)		Tafel slope ($mV{\cdot}dec^{-1}$)	Stability[a]	Electrolyte	Ref.
		At 1 $mA{\cdot}cm^{-2}$	At 10 $mA{\cdot}cm^{-2}$				
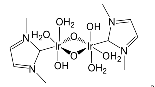 $[Ir(dmimd)(OH)(H_2O)_2(\mu\text{-}O)]_2^{2+}$	ITO nanoparticles	184	270	107	Stable until 12 h (CA)	0.1 M $KClO_4$ (pH 2)	Present work
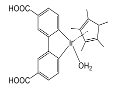 $[Ir(bpyCOOH)Cp^*(OH)_2]^{2+}$	ITO film	470[b]	-	-	25% after 13 h (CA)	0.1 M HNO_3 (pH 1)	[1]
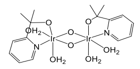 $[Ir(pyalc)(H_2O)_2(\mu\text{-}O)]_2^{2+}$	ITO nanoparticles	273	-	66	50 mV after 11 h (CP)	0.1 M KNO_3 (pH 2.6)	[2]
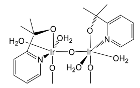 $[Ir_2(pyalc)_2(H_2O)_4\text{-}(\mu\text{-}O)]^{2+}$	ITO nanoparticles	-	320	-	-	0.1 M $HClO_4$ (pH 1.2)	[3]
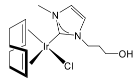 $[IrCl(cod)\{MeIm(CH_2)_3OH\}]$	reduced graphene oxides	-	538	185.5	50% after 10 min (CA)	1.0 M K-P_i (pH 7)	[4]
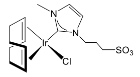 $[Ir(cod)\{MeIm(CH_2)_3SO_3\}]$	carbon nanotubes	-	438	-	66% after 1h (CA)	1.0 M K-P_i (pH 7)	[5]
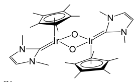 $[\{Ir^{IV}(Cp^*)(Me_2NHC)\}_2(\mu\text{-}O)_2]^{2+}$	glassy carbon	-	770	-	90% after 200 sec (CA)	0.1 M Na_2SO_4 (pH 1)	[6]
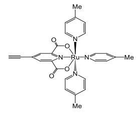 $[Ru^{II}(pdc)(pic)_3]$	glassy carbon	812[c]	-	-	-	1.0 M K-P_i (pH 7)	[7]
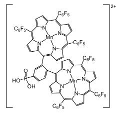 $Mn_2DP\text{-}PO_3H_2$	ITO film	470[d]	-	90	Stable until 5 h (CA)	0.025 M $Na_2B_4O_7$ + 0.1 M $NaClO_4$ buffer (pH 7.0)	[8]
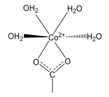 $[Co\text{-}COO]^+$	graphene	722	-	-	-	1.0 M KNO_3	[9]
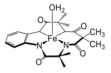 Fe-TAML	Vulcan XC-72	-	729[e]	-	Stable until 3 h (CP)	0.1 M HNO_3	[10]
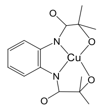 $(NMe_4)_2[Cu(L_2)]$	-	908	-	-	Stable until 3 h (CA)	0.1 M $KHCO_3$ buffer (pH 9.8)	[11]

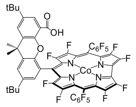 CoH$^{\beta F}$CX-CO_2H	-	740	-	120	Stable until 1 h (CA)	1.0 M K-P_i (pH 7)	[12]
IrO_x/ITO	ITO nanoparticles	-	375	-	80% after 10 h (CA)	0.1 M $KClO_4$ (pH 2)	Present work
Ir black	-	-	305	75	-	0.5 M H_2SO_4	[13]
IrO_2	-	-	305	75	-	0.5 M H_2SO_4	[13]
$IrO_x(OH)_y$	-	-	310	50	Stable until 16 h (CA)	0.5 M H_2SO_4	[14]
3R-IrO_2	-	-	188	52	Stable until 511 h (CA)	0.1 M $HClO_4$	[15]
1T-IrO_2	-	-	192	49	Stable until 126 h (CA)	0.1 M $HClO_4$	[16]
Ir nanosheets	-	-	240	49	Stable until 8 h (CA)	0.5 M H_2SO_4	[17]
Ir-IrO_x/C	-	-	198	106	Stable until18 h (CA)	0.5 M H_2SO_4	[18]
IrO_x@Ir	-	-	300	110	-	0.1 M $HClO_4$	[19]
IrNi@IrO_x	-	-	310	50	-	0.5 M H_2SO_4	[20]
Y_2IrO_7	-	-	270	50	-	0.1 M $HClO_4$ /1 M H_2SO_4	[21]
$LaLiIrO_6$	-	-	280	50	-	0.1 M H_2SO_4	[22]
Ba_2MIrO_6	-	-	330	60	Stable until 1 h (CA)	0.1 M $HClO_4$	[23]
IrO_x/$SrIrO_3$	-	-	300	40	Stable until 30 h (CA)	0.5 M H_2SO_4	[24]
Ir-$NiCo_2O_4$	-	-	240	60	Stable until 70 h (CA)	0.5 M H_2SO_4	[25]
Ir-ITO	-	-	300	46	Stable until 2h (CA)	0.1 M $HClO_4$	[26]
Li-IrO_x	-	-	270	39	Stable until 10 h (CA)	0.5 M H_2SO_4	[27]
$IrHf_xO_y$	-	-	330	50	Stable until 6 h (CA)	0.1 M $HClO_4$	[28]
IrO_2-TiO_2	-	-	255	42	-	0.1 M $HClO_4$	[29]
$W_{0.99}Ir_{0.01}O_{3-\delta}$	-	-	400	120	Stable until 0.56 h (CA)	0.5 M H_2SO_4	[30]
MnO_2	-	-	489		-	0.1 M H_2SO_4	[31]
$Ni_{0.5}Mn_{0.5}Sb_{1.7}O_y$	-	-	627		-	0.1 M H_2SO_4	[32]
Ni_2Ta	-	-	570		-	0.5M H_2SO_4	[33]
NiFeP	-	-	540		-	0.05 M H_2SO_4	[34]
1T-MoS_2	-	-	420		-	0.5 M H_2SO_4	[35]
N-WC	-	-	470		-	0.5 M H_2SO_4	[36]
CN_x	-	-	400		-	0.1 M $HClO_4$	[37]

[a]The ratio of decreased current density or increased overpotential after stability tests by chronoamperometry (CA) or chronopotentiometry (CP). [b]the overpotential (mV) was obtained at 0.01 mA·cm^{-1}. [c]the overpotential (mV) was obtained at 0.05 mA·cm^{-1}. [d]the overpotential (mV) was obtained at 0.12 mA·cm^{-1} ethe overpotential (mV) was obtained at 5 mA·cm^{-1}, 45% faradic efficiency for O_2 fthe overpotential (mV) was obtained at 0.2 mA·cm^{-1}.

Table S2. Atomic ratio of In/Sn after chronoamperometry in a 0.1M $KClO_4$ electrolyte (pH 2)

Transient time	0 min	20 min	8 h	12 h
Atomic ratio	7.82	7.61	7.08	6.66

Supporting References

[1] K. S. Joya, N. K. Subbaiyan, F. D'Souza, H. J. M. de Groot, *Angew. Chem., Int. Ed.* **2012**, 51, 9601.
[2] S. W. Sheehan, J. M. Thomsen, U. Hintermair, R. H. Crabtree, G. W. Brudvig, C. A. Schmuttenmaer, *Nat. Commun.* **2015**, 6, 6469.
[3] C. Bozal-Ginesta, R. R. Rao, C. A. Mesa, Y. Wang, Y. Zhao, G. Hu, D. Antón-García, I. E. L. Stephens, E. Reisner, G. W. Brudvig, D. Wang, J. R. Durrant, *J. Am. Chem. Soc.* **2022**, 144, 8454.
[4] B. Sánchez-Page, A. M. Pérez-Mas, M. González-Ingelmo, L. Fernández, Z. González, M. V. Jiménez, J. J. Pérez-Torrente, J. Blasco, G. Subías, P. Álvarez, M. Granda, R. Menéndez, *J. Organomet. Chem.* **2020**, 919, 121334.
[5] J. Nieto, M. V. Jiménez, P. Álvarez, A. M. Pérez-Mas, Z. González, R. Pereira, B. Sánchez-Page, J. J. Pérez-Torrente, J. Blasco, G. Subias, M. a. Blanco, R. Menéndez, *ACS Appl. Energy Mater.* **2019**, 2, 3283.
[6] D. G. H. Hetterscheid, C. J. M. van der Ham, O. Diaz-Morales, M. W. G. M. Verhoeven, A. Longo, D. Banerjee, J. W. Niemantsverdriet, J. N. H. Reek, M. C. Feiters, *Phys. Chem. Chem. Phys.* **2016**, 18, 10931.
[7] L. Tong, M. Göthelid, L. Sun, *Chem. Commun.* **2012**, 48, 10025.
[8] E. A. Mohamed, Z. N. Zahran, Y. Naruta, *J. Catal.* **2017**, 352, 293.
[9] X. Zhou, T. Zhang, C. W. Abney, Z. Li, W. Lin, *ACS Appl. Mater. Interfaces* **2014**, 6, 18475.
[10] E. L. Demeter, S. L. Hilburg, N. R. Washburn, T. J. Collins, J. R. Kitchin, *J. Am. Chem. Soc.* **2014**, 136, 5603.
[11] M. Bera, K. Keshari, A. Bhardwaj, G. Gupta, B. Mondal, S. Paria, *Inorg. Chem.* **2022**, 61, 3152.
[12] D. K. Dogutan, R. McGuire, Jr., D. G. Nocera, *J. Am. Chem. Soc.* **2011**, 133, 9178.
[13] C. Rakousky, M. Shviro, M. Carmo, D. Stolten, *Electrochim. Acta* **2019**, 302, 472.
[14] C. Massué, X. Huang, A. Tarasov, C. Ranjan, S. Cap, R. Schlögl, *ChemSusChem* **2017**, 10, 1958.
[15] Z. Fan, Y. Ji, Q. Shao, S. Geng, W. Zhu, Y. Liu, F. Liao, Z. Hu, Y.-C. Chang, C.-W. Pao, Y. Li, Z. Kang, M. Shao, *Joule* **2021**, 5, 3221.
[16] Q. Dang, H. Lin, Z. Fan, L. Ma, Q. Shao, Y. Ji, F. Zheng, S. Geng, S.-Z. Yang, N. Kong, W. Zhu, Y. Li, F. Liao, X. Huang, M. Shao, *Nat. Commun.* **2021**, 12, 6007.
[17] B. Jiang, Y. Guo, J. Kim, A. E. Whitten, K. Wood, K. Kani, A. E. Rowan, J. Henzie, Y. Yamauchi, *J. Am. Chem. Soc.* **2018**, 140, 12434.
[18] L. Zu, X. Qian, S. Zhao, Q. Liang, Y. E. Chen, M. Liu, B.-J. Su, K.-H. Wu, L. Qu, L. Duan, H. Zhan, J.-Y. Zhang, C. Li, W. Li, J. Y. Juang, J. Zhu, D. Li, A. Yu, D. Zhao, *J. Am. Chem. Soc.* **2022**, 144, 2208.
[19] Y.-T. Kim, P. P. Lopes, S.-A. Park, A. Y. Lee, J. Lim, H. Lee, S. Back, Y. Jung, N. Danilovic, V. Stamenkovic, J. Erlebacher, J. Snyder, N. M. Markovic, *Nat. Commun.* **2017**, 8, 1449.
[20] H. N. Nong, T. Reier, H.-S. Oh, M. Gliech, P. Paciok, T. H. T. Vu, D. Teschner, M. Heggen, V. Petkov, R. Schlögl, T. Jones, P. Strasser, *Nat. Catal.* **2018**, 1, 841.

[21] D. Lebedev, M. Povia, K. Waltar, P. M. Abdala, I. E. Castelli, E. Fabbri, M. V. Blanco, A. Fedorov, C. Copéret, N. Marzari, T. J. Schmidt, *Chem. Mater.* **2017**, 29, 5182.
[22] A. Grimaud, A. Demortière, M. Saubanère, W. Dachraoui, M. Duchamp, M.-L. Doublet, J.-M. Tarascon, *Nat. Energy* **2016**, 2, 16189.
[23] O. Diaz-Morales, S. Raaijman, R. Kortlever, P. J. Kooyman, T. Wezendonk, J. Gascon, W. T. Fu, M. T. M. Koper, *Nat. Commun.* **2016**, 7, 12363.
[24] L. C. Seitz, C. F. Dickens, K. Nishio, Y. Hikita, J. Montoya, A. Doyle, C. Kirk, A. Vojvodic, H. Y. Hwang, J. K. Norskov, T. F. Jaramillo, *Science* **2016**, 353, 1011.
[25] J. Yin, J. Jin, M. Lu, B. Huang, H. Zhang, Y. Peng, P. Xi, C.-H. Yan, *J. Am. Chem. Soc.* **2020**, 142, 18378.
[26] D. Lebedev, R. Ezhov, J. Heras-Domingo, A. Comas-Vives, N. Kaeffer, M. Willinger, X. Solans-Monfort, X. Huang, Y. Pushkar, C. Copéret, *ACS Cent. Sci.* **2020**, 6, 1189.
[27] J. Gao, C.-Q. Xu, S.-F. Hung, W. Liu, W. Cai, Z. Zeng, C. Jia, H. M. Chen, H. Xiao, J. Li, Y. Huang, B. Liu, *J. Am. Chem. Soc.* **2019**, 141, 3014.
[28] F. Zhao, B. Wen, W. Niu, Z. Chen, C. Yan, A. Selloni, C. G. Tully, X. Yang, B. E. Koel, *J. Am. Chem. Soc.* **2021**, 143, 15616.
[29] E. Oakton, D. Lebedev, M. Povia, D. F. Abbott, E. Fabbri, A. Fedorov, M. Nachtegaal, C. Copéret, T. J. Schmidt, *ACS Catal.* **2017**, 7, 2346.
[30] S. Kumari, B. P. Ajayi, B. Kumar, J. B. Jasinski, M. K. Sunkara, J. M. Spurgeon, *Energy Environ. Sci.* **2017**, 10, 2432.
[31] A. Li, H. Ooka, N. Bonnet, T. Hayashi, Y. Sun, Q. Jiang, C. Li, H. Han, R. Nakamura, *Angew. Chem., Int. Ed.* **2019**, 58, 5054.
[32] I. A. Moreno-Hernandez, C. A. MacFarland, C. G. Read, K. M. Papadantonakis, B. S. Brunschwig, N. S. Lewis, *Energy Environ. Sci.* **2017**, 10, 2103.
[33] J. S. Mondschein, K. Kumar, C. F. Holder, K. Seth, H. Kim, R. E. Schaak, *Inorg. Chem.* **2018**, 57, 6010.
[34] F. Hu, S. Zhu, S. Chen, Y. Li, L. Ma, T. Wu, Y. Zhang, C. Wang, C. Liu, X. Yang, L. Song, X. Yang, Y. Xiong, *Adv. Mater.* **2017**, 29, 1606570.
[35] J. Wu, M. Liu, K. Chatterjee, K. P. Hackenberg, J. Shen, X. Zou, Y. Yan, J. Gu, Y. Yang, J. Lou, P. M. Ajayan, *Adv. Mater. Interfaces* **2016**, 3, 1500669.
[36] N. Han, K. R. Yang, Z. Lu, Y. Li, W. Xu, T. Gao, Z. Cai, Y. Zhang, V. S. Batista, W. Liu, X. Sun, *Nat. Commun.* **2018**, 9, 924.
[37] K. Mamtani, D. Jain, D. Dogu, V. Gustin, S. Gunduz, A. C. Co, U. S. Ozkan, *Appl. Catal., B* **2018**, 220, 88.
